# Reinventing malaria control: hierarchical intervention strategies for African settings based on the microbiota-barrier-innate immunity axis

**DOI:** 10.3389/fimmu.2026.1782402

**Published:** 2026-03-18

**Authors:** Yan Zhu, Shengkao Wu, Yanwei Qi, Junjie Wang

**Affiliations:** 1 The Second School of Clinical Medicine, Guangzhou Medical University, Guangzhou, China; 2 Department of Pathogenic Biology and Immunology, School of Basic Medical Sciences, Guangzhou Medical University, Guangzhou, Guangdong, China; 3 Department of Infectious Diseases, The Second Affiliated Hospital, Guangzhou Medical University, Guangzhou, China

**Keywords:** gut microbiota dysbiosis, intervention pyramid, malaria, microbiota-barrier-innate immunity axis, short-chain fatty acids

## Abstract

Malaria control is challenged by parasite drug resistance and inadequate vaccine protection. Although mosquito gut microbes regulate *Plasmodium* development, integrated analyses of microbiota-*Plasmodium*-host interactions are lacking. Innate immunity imbalance is the primary driver of disease pathogenesis. This review proposes a unified “microbiota-barrier-innate immunity” axis hypothesis. Under physiological conditions, gut microbiota modulates host innate immunity via metabolites and maintains barrier integrity. However, *Plasmodium* infection disrupts this axis, causing microbiota imbalance, metabolic derangement, intestinal barrier leakage. The ensuing innate immune dysfunction exacerbates the pathology of malaria. Based on this, we constructed a hierarchical three-tier foundational-targeted-precision intervention pyramid tailored to diverse settings, particularly African regions, with endogenous and technological feasibility. This framework, progressing from foundational nutritional regulation and targeted microbial intervention to precision bioengineering, provides an implementable malaria control strategy for resource-limited African areas, addressing drug resistance and vaccine shortages. It offers a translatable eco-immunological approach to accelerate global malaria elimination.

## 1 Introduction

Malaria thus constitutes a considerable danger to human existence as a widespread infectious disease, which is endemic to tropical and subtropical regions. The continuance in its control is still beset with a spectral number of structural obstructions, and the disease has not yet been suppressed. Global malaria control still faces a very difficult situation. In 2023, the number of cases moved in the opposite direction of the expected decline and reached 263 million, with about 597,000 deaths; this mortality level was far above international targets. The disease burden is also unevenly distributed, with Africa accounting for 94% of cases and 95% of deaths worldwide (1).Clinically the disease has three forms, namely, severe or complicated, mild or uncomplicated, and asymptomatic (2). The host’s response to infection varies significantly and is influenced by multiple factors. These include parasite protein genetics, co-infections, comorbidities, ethnic background, geographical location, and microbiome composition, as well as the host’s nutritional and genetic background (3, 4). During the progression from infection to the blood stage of the disease, the host’s immune responses are sequentially initiated. This process begins with physical barriers such as the skin, followed by innate immunity mediated by cells like dendritic cells. Ultimately, it develops into adaptive immunity such as the production of cytophilic antibodies (5).

### 1.1 Bottlenecks in malaria control

The transmission of *Plasmodium vivax* is highly region-specific. A large-scale genomic study in Brazil supports this. South American parasite populations form a distinct global subgroup. Within Brazil, the parasites are highly differentiated. They form at least seven genetic clusters. The study also identified mutations and selection signals specific to or common in Brazil. These signals are concentrated in two gene groups. One group relates to antimalarial drug sensitivity, such as *pvmdr1* and *pvdfr-ts*. The other group relates to the mosquito transmission stage, such as *pvcrmp3*, *pvp47*, and *pvp48/45 (*6). This finding indicates that the *Plasmodium* populations in Brazil have a unique evolutionary trajectory. The regional drug resistance-related mutations and local mosquito-borne adaptive evolution collectively highlight the limitations of strategies based on uniformity. Moreover, the effect of climate change on transmission may be contrary to conventional thinking. In regions of Africa and Asia where transmission is intense, when temperature rises above an optimal level the vectorial capacity of Anopheles mosquitoes may be sharply curtailed or completely destroyed (7). This indicates that global warming will not lead to a uniform acceleration of malaria transmission. Instead, due to the heterogeneity of climatic conditions across different subregions within areas of high transmission, its impact on malaria spread is not uniform. It is more likely to reshape geographic distribution patterns with differential effects.

Apart from ecological factors, the emergence and spread of multidrug resistance in *Plasmodium* parasites to the key drug artemisinin and its combination therapies has led to a steep decline in the cure rates of traditional artemisinin-based Combination Therapy. In response to the known resistance crisis in hotspots such as Cambodia and Thailand, researchers have developed novel approaches like Triple artemisinin-based Combination Therapies (TACTs) to combat resistance. These therapies work together effectively. They work together effectively through multiple mechanisms, including inhibition of nucleic acid synthesis, disruption of heme detoxification, and generation of toxic free radicals. Each drug uses a unique mechanism. Even if the parasite develops resistance to a single drug, it struggles to simultaneously evade the concerted action of three distinct mechanisms. TACTs can increase the cure rate to over 95% and effectively delay the development of resistance. However, these new solutions have also led to secondary bottlenecks. Although TACTs are well-tolerated, they suffer from mild QTc prolongation and increased vomiting. Additionally, the problems with TACT are its high cost, unreliable supply, and ethical issues that hamper their availability and widespread use in limited-resource environments (8), and place the global malaria control project in a vicious cycle between drug resistance and adequate countermeasures.

RTS,S/AS01, recently approved by the WHO for use in African children, represents the current state of the art in this field. The core component is a virus-like particle that expresses the 19 NANP repeats of the circumsporozoite protein of *Plasmodium* falciparum and the C-terminal region of the 3D7 isolate, fused to hepatitis B surface antigen. In addition, the vaccine contains unmodified HBsAg and the adjuvant AS01E (9). The vaccine was shown to provide about 50% protection for up to 14 months after vaccination in African children in phase 3 trials, but this protection waned with time (10, 11). Also, the IFN-γ response induced by the vaccine mainly comes from NK cells rather than T cells, suggesting that CD8+T cells may not be fully activated. Furthermore, it may also fail to fully mobilize and activate NK cells and other lymphocytes with innate-like characteristics over a long period of time, whereas these cells play a crucial role in controlling early intrahepatic stage infection and establishing a rapid immune response defense (12, 13).

Recent research further challenges the traditional paradigm focused solely on total antibody titers. Protection against malaria shows no correlation with total IgG levels but is significantly associated with specific IgM and IgG subtypes targeting α-galactose (14). Antibodies targeting a new β-ctCSP epitope have also been shown to be present in subjects receiving the RTS,S/AS01 vaccine that cause broad reactivity against different field-isolated strains and produce an inhibition of infection (15). This evidence collectively indicates that future vaccine design strategies must move beyond a single-antigen-epitope approach and instead aim to shape an innate immune landscape based on the specific antibody subtypes. This direction provides a key path for improving the breadth and persistence of existing vaccine protection.

### 1.2 Focusing on the central role of innate immunity

In the deep mechanism of the bottleneck mentioned above, innate immunity plays a crucial role. It not only serves as the first line of rapid defense, but also plays a crucial role in shaping the pattern of anti-malarial immune responses. The traditional binary view of “pathogen-host” has been insufficient to better interpret the pathology and immune dysregulation of malaria. Recent studies have shown that gut microbiota plays a critical role in this process (16, 17). Due to differences in the abundance of *Lactobacillus* and *Bifidobacterium* in mice, genetically identical mice from different sources exhibited significantly divergent parasite loads and mortality rates after *Plasmodium* infection (18). This phenomenon reminds us that the dynamic tripartite interaction of “host-pathogen-microbiota” should be included in the research core.

At the host level, genetic polymorphism of C3, the main agent of innate immunity, can influence the risk of children developing severe malarial anemia (19). At the transmission stage, the newly uncovered evidence indicates that functionally intact C3 in the host is required for *Plasmodium* to infect mosquitoes and complete its life cycle successfully. C3 directly lyses the anti-malarial symbiotic bacterium *Elizabethkingia anophelis* in the mosquito midgut following its activation through the alternative pathway. *E. anophelis* prevents the development of *Plasmodium* in the midgut of the mosquito by inhibiting the conversion of the zygotes to ookinetes, and C3’s killing of these bacteria indirectly removes a development block for transmission of the parasite. On the contrary, if host C3 is deficient, or if the activation of C3 is inhibited by the factor B inhibitor LNP023, then the abundance of *E. anophelis* in the mosquito midgut increases, and transmission of the parasite to mosquitoes is heavily suppressed. Further, aberrant C3 activation rapidly reduces the efficacy of Transmission-Blocking Vaccines such as those providing immunity against Pfs25, but inhibiting C3 activation significantly enhances their transmission-blocking efficacy (20). This interaction between the *Plasmodium* parasite and the mosquito host not only expands our understanding of the ecological functions of innate immune molecules, but also reveals the unique strategy employed by *Plasmodium* to utilize the host complement system to facilitate its transmission. This characteristic further underscores the importance of microbiota in regulating C3 function.

Drawing on the preceding portrayal of bottlenecks in malaria control and core principles of the “host-pathogen-microbiota” paradigm, we put forward an integrative theoretical concept of the “microbiota-barrier-innate immunity” axis at the heart of burgeoning malaria pathology. In a healthy state, the gut microbiota secretes metabolites such as Short-chain Fatty Acids (SCFAs), which strengthen intestinal epithelial tight junctions and mucus secretion, enhancing barrier integrity. The microbiota and “barrier” work in concert as a calibration center, fine-tuning the functional states of innate immune cells such as macrophages, NK cells, and Vδ2+ γδ T cells, through key signaling pathways, to enact an effective but restrained system-wide innate immunity. During *Plasmodium* infection, the parasite first disrupts the microbiota structure, initiating dysbiosis, and second, disrupts the intestinal barrier allowing microbial translocation and inflammation to ensue. This “leakiness” interrupts the calibration signals to the innate immune and complement systems: disturbed signaling, many of the immune effects triggered at the cellular level seem to result in dysfunctional macrophages, dendritic cells, and γδ T cells, driving malaria pathogenesis and transmission. How the microbiota tunes the complement system in homeostatic regulation is thus key to comprehending the physiologic “calibration center” of this axis, aiding it’s seeming collapse entwined with malaria pathology. Guided by this integrated construct, we highlight below specific homeostatic regulatory mechanisms of this axis under healthy conditions, and the *Plasmodium*-induced disruptive forces that follows, laying theoretical groundwork for subsequent intervention strategies.

### 1.3 Review focus, conceptual framework, and scope boundaries

This review is designed to lay out, in a systematic way, how mosquito vectors and host gut microbiota participate in malaria transmission, and how these findings can be translated into practical applications. The primary focus is the malaria system transmitted by *Anopheles* mosquitoes, with particular attention to the adult mosquito midgut as a key ecological niche. This midgut environment is not only where *Plasmodium* sporogony takes place, but also where tripartite interactions among the microbiota, the parasite, and the host immune system are most directly manifested. At the same time, to maintain an integrated perspective, the human host gut microbiota is also included as part of the discussion, viewed as an important node in systemic immune regulation, thereby supporting a cross-host analytical line that follows “vector–pathogen–human.”

To address these questions in detail, the review uses a multidimensional integrative approach that combines laboratory basic research, animal models, and field epidemiology in Africa. Laboratory work helps clarify fine-scale mechanisms under controlled settings; animal models offer a reproducible way to test causal links; and field studies help assess ecological validity and translational value under real conditions. Each approach also has clear limitations: animal models cannot fully represent human-specific factors such as nutrition, coinfections, and behaviors, and observational field studies usually cannot prove causality. For this reason, when results across evidence sources do not align, they need to be interpreted critically and in an integrated manner.

For theoretical organization, the review follows “microbiota-immunity-parasite” triadic interactions as the central thread. These interactions are discussed across three complementary disciplinary dimensions. From an ecological perspective, attention is given to how community structure influences *Plasmodium* fitness; from an immunological perspective, the focus is on how the microbiota tunes innate immunity through metabolites and how *Plasmodium* can exploit these processes; and from a translational perspective, the aim is to gradually turn basic findings into implementable intervention strategies, including nutritional interventions, microbiota regulation, and bioengineering technologies, so that a precision public health system can be developed for resource-limited settings in Africa.

On this basis, the review first outlines the homeostatic regulatory mechanisms of the “microbiota-barrier-innate immunity” axis under healthy conditions; it then examines how *Plasmodium* infection disturbs this axis, producing dysbiosis, barrier leakage, and immune dysregulation; and finally, using mechanistic insights as a foundation, it proposes a three-tier intervention pyramid oriented to African field settings and discusses practical implementation routes, enabling systems, barriers, and future directions. The overall goal is to provide malaria control with a clear and workable translational path that connects theory with practice and links laboratory research to field deployment.

## 2 The “microbiota-barrier-innate immunity” axis in health

### 2.1 Gut microbiota homeostasis and the immunometabolic regulatory network

As a complex ecosystem, the structural stability of the gut microbiota is the bedrock on which gastrointestinal homeostasis and host immunity rest. *Firmicutes*, *Bacteroidetes*, *Actinobacteria* and *Proteobacteria* are the four core phyla in the gut of healthy adults (21). The main regulatory mediators is the variety of metabolites synthesized by the microbiota which together comprise the microbiota-immune chemical signaling network. Metabolites can be roughly classified by their origins into three major categories: the first is metabolites generated by fermentation or degradation of dietary fiber; the second is host-derived metabolites that have been modified by microbes; and the third is *de novo* synthesized microbial metabolites, including effector molecules recognized by the host (22). During malaria infection, these various classes of metabolites might have differential immunomodulatory effects that provide a rationale for a metabolite target database for the precise regulation of the innate immunity.

Homeostasis of gut microbiota is important for preventing aberrant activation of inflammatory pathways such as cGAS-STING. Dysbiosis may lead to immune disorders and aberrations in innate immune pathways. For example, in the STING-related infantile-onset vascular disease mouse model, the decrease of Short-chain Fatty Acid (SCFA) producing bacteria and the rise of segmented filamentous bacteria lead to the increased levels of microbially- and host-derived cyclic dinucleotides, leading to STING pathway activation and consequently exacerbated inflammatory responses (23). In addition, pathogenic DNA from the lethal malaria parasite *Plasmodium yoelii* Nigeria subspecies N67C clone activated the cGAS-STING signaling pathway in innate immunity. This activation predominantly induced late-stage IL-6 production predominantly through the MyD88-p38 axis and increased CD11b+ Ly6Chi pro-inflammatory monocytes. Both of these suppressed effective adaptive immune responses and increased host mortality. In contrast, cGAS or STING knockout mice exhibited significantly prolonged survival upon infection (24). These findings collectively establish the STING pathway’s core hub connecting malaria infection and pathological inflammation.

### 2.2 The immunomodulatory effects of short chain fatty acids

SCFAs are well-recognized microbiota-dependent metabolites produced in the gut through the fermentation of dietary fiber, principally including butyrate, propionate and acetate. As key mediators of microbiota-host communication, SCFAs fulfil numerous functions in regulating immune responses, alter intestinal integrity, and calibrate systemic immune homeostasis, principally via HDAC inhibition and GPCR engagement (25).

In the context of immune regulation, SCFAs can influence the functional polarization of innate immune cells. In the lungs, they shape the immune repertoire by activating G protein-coupled receptors including GPR43 (26). Meanwhile, the regulatory effect of SCFAs on macrophage function has also been directly observed in intestinal anti−infection immunity. The gut microbiota metabolite butyrate might strengthen the antibacterial ability of differentiating macrophages through inhibition of the histone deacetylase 3 (HDAC3) enzyme (27). In addition to this, SCFAs play a major role in the orchestration of intestinal circadian rhythms and epithelial homeostasis. Intestinal organoid study uncovered that SCFAs generated by specific gut microbes like *Clostridium* and *Parabacteroides goldsteinii* can substantially shift the phase of circadian oscillations in epithelial cells through HDAC inhibition (28), highlighting the direct regulatory capacity of microbial metabolites on host physiological rhythms.

Clinical intervention studies corroborate the pivotal involvement of SCFAs here, with the Mediterranean diet reported to significantly enhance fecal content of propionate and butyrate, accompanied by improved intestinal permeability markers (29). Therefore, in the context of malaria, maintaining sufficient SCFAs levels may be beneficial for modulating innate immune responses and increasing resistance of the host to *Plasmodium* infection.

### 2.3 Maintenance of barrier integrity

The healthy intestinal barrier is a multi-layered and highly coordinated defense system that not only provides physical isolation but also has an active immune surveillance function, collectively maintaining host homeostasis. The physical barrier is mainly composed of intestinal epithelial cells and tight junction proteins between them. A mucus layer secreted by goblet cells covers the surface, effectively separating the gut microbiota from the epithelial surface. Furthermore, complete barrier function also includes its superficial immune barrier. In particular, secretory immunoglobulin A secreted by the mucosal immune system plays an irreplaceable role in maintaining microbiota homeostasis and preventing pathogen adhesion (30).

Clinical data collected from patients who underwent splenectomy and patients with variant immunodeficiency diseases provide strong evidence for the role of the immune barrier. The absence of IgM memory B cells leads to a deficiency in the secretion of SIgA in mucosal lymphoid tissues; function of the mucosal barrier is compromised, which allows pathogens to cause more severe symptoms of infection (31). This strengthens the concept that IgM memory B cells are important for mucosal SIgA homeostasis and mucosal barrier integrity. Thus, we can hypothesize that although individuals in malaria-endemic areas have normal immunity, their mucosal B-cell function may be damaged due to malnutrition and/or repeated infections, which might further exacerbate microbial dysbiosis and render them susceptible to malaria.

### 2.4 Calibration and priming of innate immune cells

The host's innate immune system can respond quickly after sensing pathogen invasion. For example, during *Plasmodium yoelii* infection, infection activates macrophages and polarizes them towards an M1 phenotype, aiding their phagocytic function. This was observed to correlate with a state of TLR7 upregulation and macrophage STAT3 activation, which the authors report to be mediated by the action of the TLR7-STAT3 pathway. Depleting macrophages via clodronate liposomes, the authors found from their study that the host suffers more severe parasitemia and shows greatly compromised adaptive immune responses (32). Taken together, these results suggest that the TLR7-STAT3 signaling pathway, by influencing the functional activation of macrophages at an early stage of malaria infection, is required for the subsequent initiation of an adaptive immune response. This demonstrates a positive role of this pathway in early host defence against infection.

Beyond affecting myeloid immune cells through similar modalities, microbial signals functionally shape innate-like lymphocytes as well. One of the primary protective mechanisms provided by natural killer (NK) cells in healthy individuals is antibody-dependent cellular cytotoxicity (ADCC), and NK cells activated by appropriate IgG specific to antigens such as PfEMP1 or RIFIN are able to kill infected red blood cells in individuals from endemic areas (33). When NK cells are activated by specific antibodies against *Plasmodium* parasites, they can degranulate and release IFN-γ; this Ab-NK response has cross-strain protective ability and can enhance inhibition of parasite invasion into red blood cells (34).

Vδ2+ γδ T cells, serving as a critical bridge between innate and adaptive immunity, are functionally capable of controlling blood-stage *Plasmodium* infection in healthy individuals. The cells selectively recognize phosphoantigen-BTN3A1 complexes displayed on the surface of infected red blood cells (iRBCs) via their T cell receptor (TCR) and through the formation of an immune synapse. They then lyse the iRBCs and destroy intracellular parasites through a direct contact-dependent mechanism involving phosphoantigens and granule granzyme secretion. In parallel, Vδ2+ γδ T cells can phagocytose opsonized iRBCs via a CD16-dependent mechanism, leading to diminished parasite replication and thus facilitating a dual mechanism for protection from malaria (35). In addition, Vδ2+ γδ T cells are important in mediating the sterile immunity produced by vaccination with the PfSPZ vaccine. Furthermore, those individuals with a greatly expanded Vδ2+ γδ T cell population are more likely to benefit from vaccination. In the absence of γδ T cells, the development of CD8α+ dendritic cells in the liver of vaccinated mice is not seen (36). Thus a well-developed Vδ2+ γδ T cell repertoire is a critical determinant of whether the host immune system is able to mount an effective immune response against vaccination.

Upon repeated exposure to malaria, the innate immune system undergoes dynamic reorganization. The initial emergent response mediated by Vδ2+ γδ T cells may no longer be sustainable in quantity and therefore Vδ1+ γδ T cells play a compensatory role. As a result of the increased number, clonal selection and expansion of Vδ1+ γδ T cells, this population differentiates from a naïve state into lytic effector cells which express high levels of perforin and granzyme (37).This shift in emphasis, from Vδ2+ towards Vδ1+ subset, highlights the plasticity and adaptability of innate-like lymphocytes with respect to the establishment of long-lived anti-malarial immunity.

**Figure 1:**
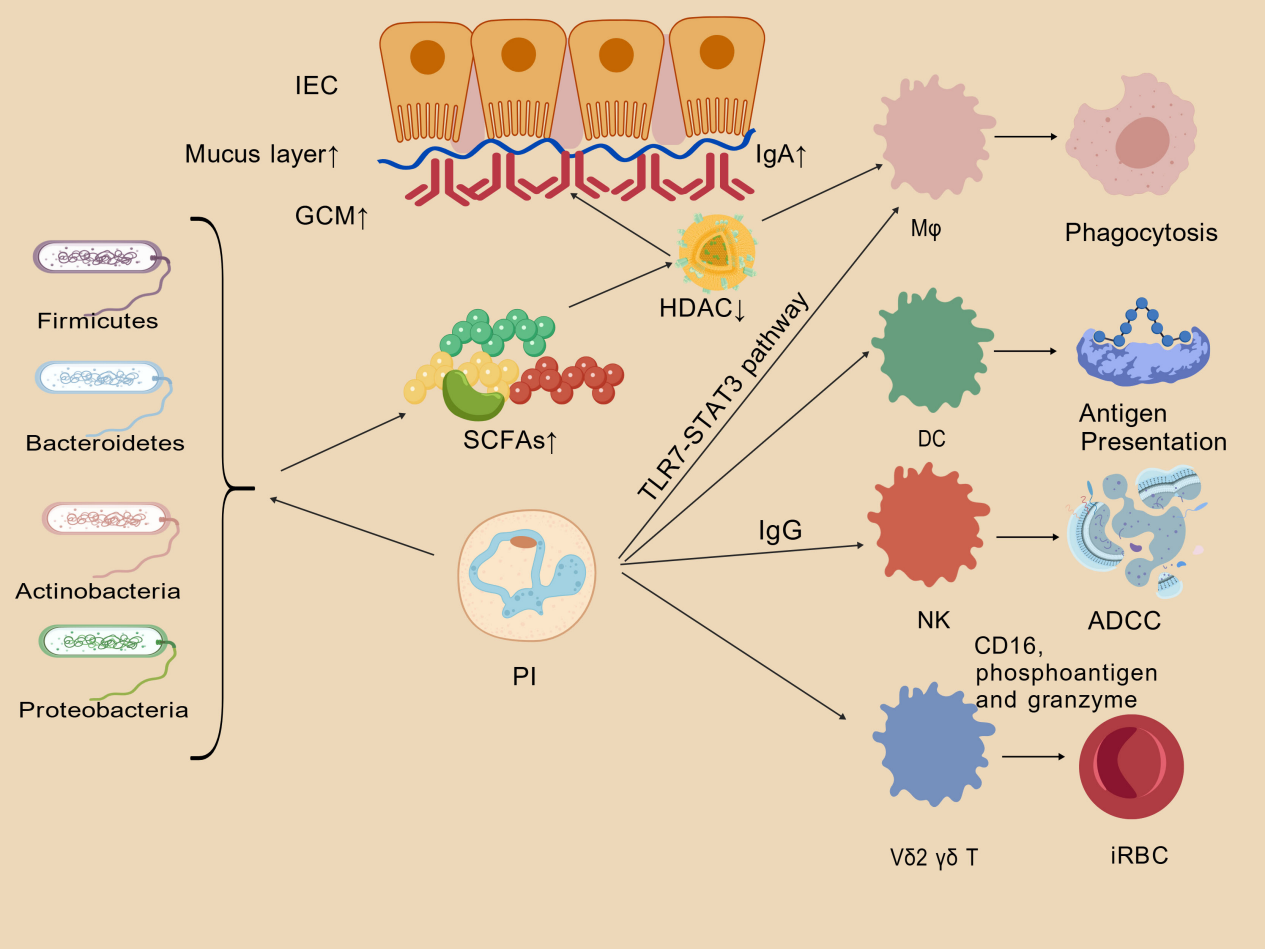
Homeostatic “core microbiota–barrier–innate immunity” axis in healthy individuals. Arrows (→) indicate induction, promotion, or enhancement; “↑”denotes an increase; “↓”denotes a decrease. IEC, intestinal epithelial cell; GC, goblet cell; Mφ, macrophage; DC, dendritic cell; NK, natural killer cell; γδ T, gamma-delta T cell; SCFAs, short-chain fatty acids; GCM, goblet cell mucins; HDAC, histone deacetylase; PI, *Plasmodium* infection; iRBC, infected red blood cell.

Therefore, under homeostatic situations, a core “microbiota-barrier-innate immunity” axis exists that ensures anti-malarial homeostasis (**Figure 1**). The molecular circuitry of this axis reveals how symbiotic bacterial metabolites fortify the barrier and tune innate immune cells. The schematic in **Figure 1** depicts the regulatory circuitry of this core homeostatic axis, whereby the core microbiota (via metabolites such as SCFAs) fortifies the intestinal epithelial barrier and tunes innate immune macrophages, NK cells and γδ T cells. This serves as an intuitive visual framework for the “microbiota-barrier-innate immunity” axis with key components drawn in.

## 3 Causal perturbation and remodeling of the “axis” by *Plasmodium* infection

### 3.1 Evidence from clinical and preclinical studies

#### 3.1.1 Human studies: linking dysbiosis and disease

The gut microbiome of malaria patients is characterized by a distinctive state of inflammatory dysbiosis. Central to this phenomenon is not the loss of microbial diversity but rather a pathological remodeling of the community structure. Strong evidence is derived from several cohort studies in Mali. A metabolomic analysis by Schmidt et al. (38), using children susceptible to malaria as the test population, showed significant elevations in fecal inflammatory markers such as deoxynosine and long-chain fatty acids, along with concurrent elevation of markers of repair such as pseudouridine and hypoxanthine.

Finding from Kodio et al. further confirmed that the gut bacterial community structure is significantly correlated with clinical malaria episodes and asymptomatic *Plasmodium falciparum* infection, with the counterintuitive observation that the higher the bacterial OTU richness, the higher the risk for such malaria episodes. During follow-up, 25 bacterial biomarkers related to malaria susceptibility or resistance were identified, including 17 susceptibility-associated taxa and 8 resistance-associated taxa (39). Most susceptibility-associated taxa belonged to pro-inflammatory genera; for instance, hypermucoviscous multidrug-resistant *Klebsiella pneumoniae ST25* can infect intestinal epithelial cells, lower cell viability, disrupt tight junctions, and upregulate the expression of multiple inflammatory factors (40). *Clostridium perfringens* beta2 toxin can trigger apoptosis in porcine intestinal epithelial cells, increase the expression of several interleukins, and weaken intestinal barrier function (41). In addition, early exposure to Enterobacteriaceae may shape the inflammatory state of the gastrointestinal tract and may modify inflammatory patterns and responsiveness to pathogens (42). In contrast, resistance-associated taxa were mainly beneficial short-chain fatty acid–producing bacteria, such as *Bifidobacterium* and *Lactobacillus (*43). Taken together, these findings imply that the high OTU richness observed during malaria infection may represent community-structure dysregulation rather than a stable healthy state.

Although current studies have described characteristic gut microbiota dysbiosis patterns linked with malaria infection, a key limitation is that clear causal relationships cannot be firmly established. The apparently paradoxical pattern of “high OTU richness accompanying high risk” suggests that the community state being measured is more likely an infection-driven inflammatory ecological remodeling, instead of a pre-existing driver of host susceptibility. Moreover, much of the literature still remains focused on species-level association testing, while possible functional mechanisms remain underexplored. Future research needs to integrate longitudinal causal experiments with functional metabolomics to define the roles of key microbial functional pathways in this context. Accordingly, microbiota-based approaches for malaria intervention should not rely on simplistic strategies to increase diversity; they need targeted therapies that can precisely reshape microbial functional ecological niches.

Current research has largely focused on specific regions in Africa, and the general applicability of the “microbiota-barrier-innate immunity” axis requires further validation across populations with varying malaria transmission intensities and ethnic backgrounds. For example, among Hmong and Karen individuals who migrated from Southeast Asia to the United States, a significant decline in gut microbiome diversity has been observed after immigration. American-associated bacterial strains and functional profiles gradually replaced native microbial communities, and this shift has been linked to an increased risk of metabolic diseases (44). The westernization of a living environment and food patterns can create a corresponding westernized gut microbiome that may, in turn, alter the host’s immune responses to multiple diseases, including malaria.

Studies from Odisha, India, report that infection with soil-transmitted helminths negatively correlates with certain gut bacterial taxa including *Lactobacillus* and *Lachnospira*. In addition, the relative abundance of *Lactobacillus* was more dependent on soil-transmitted helminth infection status than on *Plasmodium* infection status, and vice versa (45). This indicates that in the context of polyparasitism the gut microbiome could regulate susceptibility to a range of pathogens via shared immunoregulatory pathways. These findings further suggest the existence of a conserved “microbiota–barrier–innate immunity” axis that may operate across geographical locations and parasite species.

The aforementioned research conclusions are strongly influenced by geographic context. The specific relationship between microbiota OTU richness and malaria risk reported in the Malian cohort may not be reproduced in other high-transmission areas such as Southeast Asia or South America. Such heterogeneity may reflect differences in human genetic background, dominant *Plasmodium* strains, local patterns of endemic coinfections, and dietary structure. In addition, an earlier immigration study reported that after moving from Southeast Asia to the United States, the gut microbiota of Hmong and Karen groups shifted toward Westernization, which may alter immune-response profiles. Therefore, a region-specific “microbiota–disease” association pattern should not be directly extended to all malaria-endemic regions. Mechanistic models and intervention planning need to explicitly account for this geographic and population-level heterogeneity and support regionally customized research paradigms.

Extreme clinical phenotypes are important supporting evidence of the above causal chain. A case of a patient in the second trimester of pregnancy with sickle cell disease illustrates that when *Plasmodium* falciparum parasitemia is complicated by autoimmune hemolytic anemia, hemoglobin concentration drops sharply, and lactate dehydrogenase and bilirubin levels increase significantly. After the administration of antimalarial drugs, the parasites were eliminated and the hemolytic symptoms of the patient were eliminated (46). The pre-existing dysbiosis of gut microbiota and low-grade inflammation of a SCD patient were rapidly aggravated by the dual assault of pregnancy and malaria infection. The antimalarial treatment breaking this vicious circle is an indirect confirmation that the malaria parasite acted as one component of this dual assault that initiated the pathological processes.

#### 3.1.2 Animal models: causal influence of microbiota on disease outcome

The microbial community of the gut is thus itself an independent risk factor determining the outcome of infections, with transplantation experiments in germ-free mice providing direct evidence. Recipients of cecal contents from malaria-resistant mice develop parasitic infections with low burdens while recipients of microbiota from susceptible mice demonstrate high blood parasitemia (18), indicating that certain specific gut communities possess causal ability to suppress malaria.

The gut microbiota can directly prime an innate immune defense pathway independent of adaptive immunity to suppress malaria through a mechanism involving α-galactose-containing polysaccharides on the surfaces of *Plasmodium falciparum* sporozoites. The level of natural α-gal-specific IgM antibodies is significantly associated with the risk of malaria; higher levels are found in infection-naïve individuals compared to infected individuals, and in older individuals who are less likely to become infected. Mice colonized with *Escherichia coli* O86:B7 had increased anti-α-gal IgM antibodies and increased resistance to malaria. Furthermore, immunization of α-gal-deficient mice with α-galactose generated high-titer antibodies that mediated complement-dependent cytotoxicity against sporozoites immediately after mosquito bite (47).

From this, we might argue that prior to the activation of the adaptive immune responses, the gut microbiota establishes an intestinal immunological “set-point” for the host, effectively regulating natural antibodies produced by innate-like B cells and conveying susceptibility (or not) to malaria infection. This study identifies the gut microbiota as a key regulatory node of malaria severity and provides a rationale for exploring probiotic intervention and manipulation of the innate immune microenvironment.

Nonhuman primate models provide additional validation. In the longitudinal sampling of the *Plasmodium cynomolgi*-infected rhesus monkey model, a lower gut microbiota alpha-diversity at peak parasitemia and a significant increase in the relative abundance of *Proteobacteria* were observed. However, the abundances of *Lactobacillaceae*, *Prevotellaceae*, and *Spirochaetes* were decreased compared to baseline levels. But in the first recurrence stage compensatory enrichment of Lactobacillaceae was demonstrated. At the metabolomic level, significant changes in the tryptophan-kynurenine pathway, and significantly increased expression of L-tryptophan biosynthesis genes have been reported (48). This indicates that the rebounding Lactobacillaceae during recurrence can be recognized as a signal of the host’s self-limiting repair. This discovery links gut microbiota disruption to systemic innate immune inflammation. It is noteworthy that the observed compensatory enrichment of Lactobacillaceae during the first recurrence stage of the disease may be a beneficial innate immune regulatory signal initiated by the host to inhibit excessive inflammation and promote barrier repair.

In summary, clinical observations, animal transplantation experiments, and primate model studies have revealed the role of gut microbiota in malaria infection. They provide evidence from different perspectives. These include correlation, causality, and dynamic hypotheses. However, the levels and strength of these proofs differ significantly. To clearly compare these differences, main research evidence is summarized by its strength (**Table 1**).

**Table 1:** Summary of the evidence hierarchy for the association between malaria infection and gut microbiota.

Study type/model	Observation	Causal evidence	Proposed mechanism	Limitations	references
Human Clinical Studies	1. The intestinal microbiota of malaria patients presents inflammatory dysbiosis. 2. The bacterial OTU richness is positively correlated with the clinical risk of malaria. 3. Twenty-five bacterial biomarkers associated with malaria susceptibility/resistance have been identified.	Direct causal experiments are lacking. Existing studies adopt a correlational design.	The phenomenon of higher OTU richness associated with higher risk may indicate a microbiota dysbiosis state driven by the infection itself.	1. Core limitation: The correlational study design fails to establish a causal relationship. 2. Most studies only focus on species-level associations, with insufficient exploration of functional mechanisms.	(38, 39)
Animal Model (Mouse)	Mice with distinct indigenous gut bacteria exhibit different levels of parasitemia and mortality following *Plasmodium* infection.	1. Fecal microbiota transplantation (FMT) experiments directly demonstrate that the gut microbiota can causally modulate the outcome of *Plasmodium* infection. 2. Colonization with a specific strain of *E. coli* O86:B7 leads to elevated anti-α-gal antibodies and confers resistance to *Plasmodium* sporozoites.	The gut microbiota may prime the host’s early anti-malarial immune status by training innate defense pathways independent of adaptive immunity and regulating the production of natural anti-α-galactose IgM antibodies by innate-like lymphoid cells.	Findings from mouse models have not been mechanistically validated in humans. The human immune microenvironment and microbiota composition are far more complex.	(18, 47)
Animal Model (Non-human Primate)	1. The α-diversity of the intestinal microbiota decreases and the relative abundance of Proteobacteria increases at the peak of infection. 2. A compensatory enrichment of Lactobacillaceae is observed during the first relapse phase of the disease.	Longitudinal observations reveal the dynamic changes of the gut microbiota throughout the infection course, while no interventional experiments have been performed to verify its functional role.	The enrichment of Lactobacillaceae during the relapse phase may represent a host-initiated self-limiting repair signal.	The functional role of specific microbial taxa remains at the correlational and hypothetical stage.	(48)

#### 3.1.3 Discrepancies between human evidence and animal models

In the Malian cohort, the result that “the higher the bacterial OTU richness, the greater the risk of malaria episodes” stands in direct conflict with FMT findings in germ-free mice, where “a high-diversity microbiota reduces parasite burden.” This contrast indicates that diversity metrics alone may not have a consistent predictive meaning across different contexts.

Notably, just as the gut microbiome is recognized as a key mediator between the environment and human physiology, disease susceptibility, and drug responses, the genetic background and microbiome of laboratory mice are also important experimental variables that influence research outcomes. Controlling and standardizing the gut microbiome in lab mice can significantly improve the predictive power and applicability of research data when translating findings to human situations (49). This reminds us that when understanding and reconciling differences between studies, the microbiome must be considered as a core experimental variable.

Laboratory studies often start from germ-free or antibiotic-treated mice, which are essentially simplified systems without complex microbial communities. By comparison, the microbiota in field populations reflects an adaptive steady state shaped by many long-term pressures, including repeated malaria exposure, malnutrition, recurrent infections, and broad-spectrum antibiotic use. Under such conditions, increased species OTU richness could be a compensatory response to persistent inflammatory stimulation, and it may even mark the ecological expansion of pathogenic bacteria within a dysbiotic community. At present, there is still no functional evaluation framework that can reliably separate healthy high diversity from pathological high diversity, so the same diversity indicator can receive opposite health interpretations depending on the study setting.

Therefore, in field settings where infection and malnutrition coexist, the microbiota’s functional composition and its ecological relationships are more informative than simple OTU richness counts. Field interventions should not aim to raise diversity in a non-specific way; instead, they should try to restore functional balance through deep sequencing together with metabolic functional analyses.

### 3.2 Three major pathways of axis disruption by *Plasmodium*

#### 3.2.1 Dysregulation of innate immune cell function

##### 3.2.1.1 Macrophages: specific functional subversion

In malaria, the functional disorders of macrophages are directly related to determined pathologies. In the case of severe malarial anemia, macrophages that continually phagocytose the parasites present decreased expression of CD169 on the surface in consequence of losing their anchorage, hence supporting the immature red blood cells (50). Thus a vicious circle of insufficient production of red blood cells and greater destruction is established. In the case of placental malaria, although the number of macrophages and the M1/M2 ratio remain unaltered, the expression of key functional genes such as STAT-6 and ANG-1 is lessened in M2 macrophages. This disrupts the immune homeostasis of pregnancy, with consequent adverse effects on maternal health and fetal development (51). In this connection there appears to be evidence that a deficiency or more likely an imbalance in the microbial metabolites SCFAs may lead to the functional disorders in these macrophages with the consequence of exacerbating malaria pathology.

The function of the macrophage is also precisely regulated through many upstream signaling pathways, implicated in the microbiota–immune axis. The unbalanced M1/M2 ratio of macrophages can be normalized by blocking the paracrine effect of IL-6 (52). Artemisia annua acid can bind to GPR37 on the surface of macrophages, promoting their phagocytic function and enhancing their ability to clear pathogens (53). Gene variants of CSF2 affect granulocyte macrophage colony-stimulating factor expression with downstream consequences for macrophage function that ultimately influence host susceptibility to malaria and severe malarial anemia (SMA) (54). Together, these pathways constitute a functional regulatory network of macrophages, providing multiple potential targets for targeted intervention.

##### 3.2.1.2 Dendritic cells: induction of programmatic paralysis

Infection with malaria can lead to functional suppression of dendritic cells. Infected persons show a decreased frequency and an increased apoptosis of circulating CD1c^+^ myeloid dendritic cells, with surviving cells being profoundly functionally suppressed. These cells, however, show an increased capacity for tumor necrosis factor secretion, but downregulation of HLA-DR and the co-stimulatory molecule CD86 results in an impaired capacity for antigen presentation. This selective dysregulation of cytokine secretion further adds to the imbalance in immune response polarization of this disease, and makes the establishment of a targeted anti-malarial immune landscape exceedingly difficult (55).

This condition may reflect a programmed form of functional suppression that is triggered by signals from the local microenvironment. For instance, systemic inflammatory response syndrome (SIRS), whether caused by pathogen mimicry or malaria itself, can induce functional paralysis not only in conventional dendritic cells but also across successive generations of these cells. The paralyzed conventional dendritic cells have a different transcriptional and phenotypical profile which is characterized by decreased antigen capture and impaired antigen presentation and an abnormal cytokine production that prevents the proper mediation of anti-malarial immunity. Application of vaccinations like monoclonal antibodies that target one of the cDC receptors or Transforming Growth Factor-beta (TGF-β) antagonism can exhibit a partial restoration of this paralysis and associated immunosuppression (56), indicating that in both cases there is a restoration of the degree of the anti-malarial immunoprecipitation and that there is, a decrease parasitic load in the mice, proving that the programmed paralysis of the dendritic cells is reversible, thus providing an experimental basis for working towards future mechanisms based on a function restoration of dendritic cells.

##### 3.2.1.3 NK and γδ T cells: exhaustion and remodeling

Within the context of chronic infection, NK cells exhibit decreased natural cytotoxic function but an intact ability to mediate antibody-dependent cellular cytotoxicity (ADCC) (57). Areas endemic for malaria and Epstein-Barr virus (EBV) also show an increase in the frequency of functionally exhausted CD56negCD16pos NK cells in children with endemic Burkitt lymphoma (eBL) and a corresponding decrease in highly cytotoxic CD56dimCD16pos NK cells, resulting in an overall loss of cytotoxicity. Additionally, in long-term survivors of eBL, this dysfunctional NK cell phenotype can gradually normalize, suggesting the exhausted state may be reversible (58).

In high transmission environments, as children get older and have reinfections, the expansion capacity and absolute numbers of Vδ2+ γδ T cells are decreased. Submicroscopic parasitemia and immunoregulatory markers such as Tim-3 and CD57 are associated with a reduced capacity for production of the proinflammatory cytokines (59). Repeated infections also lead to a reduction in the percent and response and proliferative capacity of the Vδ2+ γδ T cells, which is associated with a reduced capacity for formation of proinflammatory cytokines. This functional exhaustion is correlated with diminished symptoms during subsequent infections, suggesting that it represents a possible disease tolerance mechanism (60). While this functional exhaustion comes at a cost to the host in acquiring clinical immunity, it also produces a therapeutic window for possibly restoring γδ T cell function by microbiota-directed interventions.

#### 3.2.2 Induction of intestinal barrier leakage

##### 3.2.2.1 Regulation by immune cells

IL-4 and IL-13 derived from eosinophils do not participate in eosinophil dependent parasitic transmission regulation, but these cytokines coordinate the protection of intestinal barrier integrity after Plasmodium yoelii infection. Specifically, IL-4/IL-13, which is dependent on eosinophils, controls mast cell activation and prevents intestinal barrier damage and bacteremia caused by infection by regulating eosinophils, macrophages, and Th17 mediated inflammation (61).

The function of the mast cell protease, Mcpt4, is more complicated. After infectious challenge, Mcpt4-deficient mice exhibited elevated intestinal TNF-α and IL-12p40 levels, indicating a more vigorous type I immune response and reduced parasitemia. However, it was also shown that these animals exhibited increased intestinal permeability and disruption of adhesion junctions including E-cadherin (62). It appears that Mcpt4 skews the immune response toward a type II immune response because of the degradation of pro-inflammatory cytokines. Therefore, it reduces the overall immune intensity systemically while indirectly helping to maintain the epithelial integrity of the intestinal barrier.

Mast cell IL-10 is also important in GI barrier protection and this has a sexually dimorphic aspect. Early in infection, mast cell number and activity in the intestine were greater in female mice than in males. With mast cell IL-10 deficiency both sexes exhibited increased intestinal permeability but by different mechanisms, the female mice exhibiting a greater localized GI inflammatory response, while in males more generalized pro-inflammatory signaling impairment was noted. This indicates that mast cell derived IL-10 protects the intestinal barrier and modifies *Plasmodium* transmission by controlling sexually dimorphic pro-inflammatory responses (63). Therefore, interventions targeting malaria-induced intestinal injury should be sex-specific to achieve a dual protective effect.

##### 3.2.2.2 Disruption of metabolic signaling

SCFA deficiency may be an important link in this chain reaction. In colitis, the SCFAs combine specifically with the Ffar2 receptor to stimulate the expression of Foxp3 and interleukin-10 (IL-10) in colonic regulatory T-cells (cTregs). This leads to an increased immunosuppressive capacity to alleviate excessive inflammation. If the Ffar2 receptor is absent, both the regulatory effect SCFAs have on cTregs and the protective effect they exhibit on the intestinal barrier are completely abolished (64).

In ulcerative colitis, butyrate can also promote the development of M2 macrophages. These macrophages, by releasing WNT signaling molecules and activating the ERK1/2 pathway, increase the expression of the goblet cell marker gene SPDEF and Mucin-2, resulting in increased thickness of the intestinal mucus layer. If WNT secretion or ERK1/2 is inhibited, the ability of M2 macrophages to amplify goblet cell function is markedly reduced. Likewise, the adoptive transfer of M2 macrophages can repair goblet cells and restore mucus after dextran sulfate sodium-induced injury (65).

These protective mechanisms revealed in the context of intestinal inflammation may have universality and are of great significance for understanding the intestinal pathology of malaria. In malaria infection, the significant reduction in SCFA production due to dysbiosis of the gut microbiota prevents the effective activation of the protective signaling pathways mentioned above due to the lack of ligands. The silence of Ffar2 signal indicates impaired Treg mediated immune suppression and epithelial tight junction maintenance function; Meanwhile, polarization inhibition of M2 macrophages leads to weakened WNT/ERK signaling and decreased ability to repair the mucus layer. Therefore, malaria infection may deplete SCFAs while disrupting immune regulatory barriers and physical mucosal barriers, forming a self-reinforcing vicious cycle.

#### 3.2.3 Distant organ injury

Neurological damage in cerebral malaria involves complex inflammatory processes. On one hand, it has been demonstrated that immune complexes formed by anti-malarial antibodies generated by the infection itself are able to cause cerebrovascular pathology (66). On the other hand, it has been elucidated in models such as inflammatory bowel disease that the breach of the intestinal barrier leads to the translocation of microbes or antigens into the blood, which in turn form circulating immune complexes. These complexes have been shown to deposit in distant organs and trigger inflammation in other diseases such as arthritis via pathways such as complement activation (67). It is thus speculated that in cerebral malaria, intestinal-derived antigens may participate in the formation of additional complexes, potentially exacerbating complement activation, systemic inflammatory load, and vascular damage in the brain. However, the specific role of this “gut-brain axis” immune mechanism in cerebral malaria remains to be validated by direct experimental evidence.

In C57BL/6 susceptible mice infected with *Plasmodium berghei* ANKA, the gut microbiota may play a role in cerebral inflammation by modulating the pro-inflammatory response of glial cells. Furthermore, antibiotic- or probiotic-induced dysbiosis contributes to preventing cerebral malaria (68). Meanwhile, systemic metabolic dysregulation is associated with cerebral malaria in children. Concentrations of kynurenine and kynurenic acid (KYNA) in cerebrospinal fluid not only reflect acute disease severity but also correlate significantly with long-term neurologic sequelae. Higher levels of kynurenine are correlated with longer coma duration, while in children aged over five years, these levels are also correlated with acute attention deficits and long-term cognitive decline (69). While KYNA has the potential to be an endogenous neuroprotective agent, conferring certain protective capacity (70), it also demonstrates its double-edged nature in that regard as well as possible functional conduits to pathology. This metabolic insult might be viewed as an additional assault on the host’s defenses, compounding the effects of intestinal barrier disruption and contributing to a vicious cycle of multi-organ dysfunction.

Equally important to the battle against malaria as a site of immunity, the immune metabolic dysregulation that takes place in the spleen represents a site of systemic dysfunction. Upon stimulation with interferon-γ, monocyte-derived dendritic cells (MODCs) in the spleen undergo metabolic reprogramming resulting in metabolic dysregulation whereby glycolysis is upregulated and there is excessive accumulation of itaconate, a metabolic intermediate of the Krebs cycle, which aggravates the activation of the STING-IRF3/IRF7 signaling axis. The activation of this pathway promotes the expression of the immune checkpoint molecule PD-L1 on MODCs, which in turn suppresses the activation and anti-malarial effector function of CD8^+^ T cells (71). This process inhibits the important anti-malarial adaptive immune response in the spleen, thus impairing the host’s ability to clear the pathogen. Although the study did not directly explore the role of gut microbiota, this metabolic immune pathway suggests that metabolites derived from gut microbiota, such as SCFA, may affect the expression of immune checkpoint molecules and downstream T cell responses by regulating the metabolic status of splenic dendritic cells.

## 4 Hierarchical intervention strategies based on “axis” repair

### 4.1 A Three-tiered intervention pyramid

#### 4.1.1 Foundational tier

The purpose of this tier is to create an improved innate immune microenvironment favoring anti-malarial responses through daily diet. An observational study conducted in Rwanda showed that residents of the Western province had significantly lower dietary fiber intake compared with other regions, and this group exhibited distinct gut microbiota beta diversity with a converging distribution pattern (72). Such data indicate that dietary habits are one of the major determinants causing differences in microbial community structure.

The biochemical evidence from intervention studies supports the mechanistic basis of this intervention. The product inulin stimulates γδ T cells and the IL-22 signaling pathways to promote the proliferation of intestinal stem cells. This, in turn, results in crypt deepening and consequent elongation of the colon, as well as remodeling of epithelial homeostasis (73). The fluorescein isothiocyanate dextran detection assays were utilized in this study to further quantitatively verify the barrier-repairing effect of the product inulin. Increased gut permeability was found in the low-fiber diet mice as evidenced by diffusion of the fluorescent probe to the liver. The group in which the product inulin was supplemented, on the other hand, produced strong fluorescent signals in the gut along with low-fluorescent signals in plasma (74). The efficacy of primary interventions ultimately depends on careful application tailoring to population-specific characteristics. In practical application, dietary treatment must account for variations in the population. In the malaria endemic zones of Ghana the factors associated with risk of anemia in boys were the consumption of overweight and flour products, while the supplementation with a heme-iron and vitamin C-rich diet reduced anemia in females by 22-50% (75). If the gender parity is intended to hold practical import, the test of its efficiency and permanence in larger populations must be awaited.

By inducing metabolic reprogramming and developmental arrest in *Plasmodium*, a ketogenic diet or the ketone body β-hydroxybutyrate enhances host resistance to malaria, revealing the potential of dietary strategies targeting metabolism in combating malaria infection (76). At the same time, inulin-propionate ester is apparently efficacious in improving the insulin resistance and inflammation (77). Hence foundational interventions can be applied in the future to novel dimensions of metabolic regulation, developing innovative techniques for malaria patients suffering from concurrent metabolic abnormalities. However, the relevant research so far has been limited to animal studies and short-term clinical trials, and their synergistic effect with traditional antimalarial measures is still not understood.

#### 4.1.2 Targeted tier

This layer builds upon the advantageous ecological foundation created in the foundational layer, in that it utilizes supplementation with defined bacterial strains or their compounds to fine-tune innate immunity with precision and specificity.

Field interventions in Africa often suffer from a lack of cold chain infrastructure. Therefore, the development of thermostable formulations is a prerequisite for technological translation. Lactate from the host can be selectively utilized by *Akkermansia muciniphila* via monocarboxylate transporters (78). It has been postulated that co-encapsulation of a thermotolerant *Akkermansia* strain with lacto-oligosaccharides may facilitate a more stable and persistent intervention, further removing the constraint of a cold chain. The formulation may be further explored for other thermotolerant strains, potentially leading to a range of shelf-stable probiotics being rendered shelf-stable through the matching of a strain for a substrate. This logistical simplification will reduce costs, making interventions accessible at the primary health care level.

Certain strains have demonstrated potential antimalarial effects in animal models. *Lactobacillus fermentum YZ01*, obtained from traditionally fermented dairy products, not only increases the intestinal lactobacilli counts in male BALB/c mice to directly strengthen the intestinal microecological barrier but also delays the onset of parasitemia and improves the survival of the host through modulation of the IgM antibody levels and IL-4/IFN-γ cytokine balance (17). Although it has enlightening significance, indicating that future malaria prevention and control interventions may focus more on strains with clear characteristics rather than generalizing to probiotic categories, this idea still needs to be further validated in human trials and different epidemiological contexts.

As among the most significant strategies from this Tier II designation, the clinical translation of coadministration of probiotics with first-line antimalarials suffers from limitations. No significant effect on the alpha/beta diversity or relative bacterial abundance of the mouse gut microbiota was observed upon administration of WHO-recommended doses of common ACT regimens such as artesunate-amodiaquine and artemether-lumefantrine, but only suggestive of minimal non-sustained effects (79). However, the combination of *Lactobacillus* and chloroquine demonstrated a significantly greater suppressive capacity against *Plasmodium* than either single therapy. Significant reductions in parasitic load in the mouse blood resulted from *Lactobacillus* alone, however, and this was greatly improved when used with chloroquine achieving complete suppression of parasitemia (80). This addresses certain theoretical concerns regarding the co-administration of drugs and probiotics.

In promoting combination strategies, strict adherence to the standard therapeutic dosage of antimalarials is essential to avoid the risks of sub-therapeutic dosing. Studies using the ICR mouse model infected with *Plasmodium berghei* ANKA revealed that chloroquine doses under 20 mg/kg resulted in treatment failure and parasite recrudescence. In doses of 5 mg/kg to 10 mg/kg, parasitemia on rebound after treatment occurred, severe histopathological damage to the liver and spleen, as well as immunological dysregulation. The 1 mg/kg dosage group reflected no significant difference in efficacy compared with the blank control group (81). Chloroquine must therefore adhere strictly to the effective therapeutic dosage, thus assuring that the drug, first and foremost, achieves its core parasiticidal effect, thus laying the proper groundwork for the probiotic adjunctive modulation.

The effect of antibiotic and antimalarial usage on the microbiota is species- and context-dependent. Antibiotic treatment can reduce the bacterial load of the midgut microbiota in Anopheles darlingi (82). Conflict with this finding arises in studies in humans and animal models. For example, in infants in Kenya, artemether-lumefantrine treatment did not lead to a significant disturbance of the microbiota (83).

These apparently conflicting findings highlight how complex it is to translate conclusions from laboratory systems into real-world settings. First, mice and humans differ in basic physiology, immune-system development, and baseline gut microbiota composition, so the strong microbial remodeling seen in animal models may not appear in humans in the same form. Second, broad-spectrum antibiotics remove bacteria in a non-selective manner, and the resulting disruption of microecology is much larger than what is caused by targeted antimalarial drugs. For these reasons, before such approaches are considered for resource-limited African field settings, they should be tested in rigorously designed and well-executed human trials. Applying animal-model logic directly to use broad-spectrum antibiotics in humans could harm the intestinal barrier, raise the risk of opportunistic infections, and accelerate antibiotic resistance, among other negative outcomes. This also shows that targeted-layer interventions must proceed through a strict translational route, moving from mechanism to population-level efficacy evaluation.

Notably, the focus of targeted interventions should not only be on live probiotics. Plant extracts or plant-derived constituents may also modulate the immune system. Research indicates that GP primarily alleviates immunosuppression and intestinal damage by remodeling the gut microbiota. This reconstructed microbial community enhances host immunity by promoting the production of immune factors and short-chain fatty acids. Crucially, fecal microbiota transplantation experiments confirmed that the microbiota reshaped by GP is sufficient to mediate these immunoenhancing effects, highlighting the gut microbiome as a key pathway through which GP exerts its functions (84). Recent studies on traditional decoctions of papaya leaves have led to broader perspectives. For instance, a combination of active components such as caffeoyl derivatives and flavonol glycosides and artesunate, appears to increase significantly the efficiency of parasite clearance and bring about protection from recrudescence (85). In terms of stability and storage and transport, materials of plant-origin in standardized preparations possess advantages inherent to their physiological makeup over live bacterial preparations and over imported standardized prebiotics. This leads to their suitability in the African situation, where resources are limited. The development of the types of intervention discussed here from locally obtainable herbally derived resources will open up a potentially low-cost avenue for targeted interventions.

#### 4.1.3 Precision tier

Various engineered symbiotic microorganisms have shown their significant potential in the laboratory. *Wickerhamomyces anomalus WaF17.12* is able to secrete a particular killer toxin, which specifically inhibits the growth of *Plasmodium* in the early sporogonic stages in the midgut of *Anopheles stephensi*. It is able to decrease the blood-stage parasitemia in the host, and preliminary safety testing on mouse cell lines showed no side effects (86). Both wild type and engineered forms of *Enterobacter cloacae* in *Anopheles stephensi* midgut are effective infection barriers against *Plasmodium berghei*. The engineered strains, GFP-D and S-HasA, caused oocyst inhibition rates exceeding 92% (87). These studies suggest that genetic engineering can functionally enhance natural symbiotic bacteria and provide a reference research paradigm for the modular design of engineered bacteria.

Another interesting strategy is the introduction of exogenous bacteria through behavioral intervention. Sugar meals containing *Enterobacter* Esp_Z directly inhibit ookinete maturation, achieving approximately 45–65% suppression of *Plasmodium* development with little or no effect on mosquito health. Esp_Z produces reactive oxygen species and interferes with antioxidant pathways such as the thioredoxin system. This low virulence toward the host is significant because it allows the transmission of strains within mosquito population groups. Genomic sequencing and transcriptomic analysis of Esp_Z have been successful in identifying candidate genes involved in bacterial selection. These genes are better adapted to metabolic processes in the mosquito midgut environment, allowing for easier colonization, and for alteration in oxidative status of the surrounding environment of the bacteria (88). This discovery provides potential molecular targets and theoretical clues for further optimizing the midgut colonization ability of the Esp_Z strain. Future research should also aim for a better understanding of the mosquito-microbe symbiotic relationships. Minimal genetic engineering methods could then be employed to achieve ecologically friendly intervention methods in the future.

Another way of modifying gut microbiota is through Fecal Microbiota Transplantation (FMT), which is worthy of discussion concerning its use. There is no current evidence for FMT in malaria treatment per se, but there is evidence that it can improve the epithelial barrier of the intestine in several models of colitis by downregulation of the NF-κB pathway and increase the amount of SCFA producers (89). After C. difficile infection, FMT treatment can improve the OTU richness and diversity of fungal taxa, with efficacy depending on the fungal colonization ability of the donors used (90). The above findings may provide mechanistic references for understanding the potential value of fecal microbiota transplantation in malaria treatment. One application direction worth exploring is that fecal microbiota transplantation may be explored as an adjuvant therapy strategy in individuals with severe malaria, leading to severe depletion of gut microbiota, and poor response to conventional treatment.

The rapid advance of precision intervention technologies, including gene editing tools represented by clustered regularly interspaced short palindromic repeats/CRISPR/Cas9, provides possibilities for malaria control and prevention. This technology not only enables the generation of transgenic *Plasmodium* for pathogen characterization, but also enables the rapid spread of anti-malarial genes in mosquito vector populations or the induction of a sterile phenotype via gene drive systems, thus directly blocking malaria transmission at its source (91). However, the power of this technology is matched by the risks that it entails. Released genes could appear in wild populations and continue to survive and spread in wild populations, leading to profound and perhaps irreversible consequences for ecosystems. Thus, the progress in the research and development of such technologies should go in parallel with the setting up of the appropriate biosafety assessment framework and international regulatory consensus, taking due account of the enormous potential for the technologies against the possible risks, with a prudent approach.

#### 4.1.4 Public health pyramid

**Figure 2:**
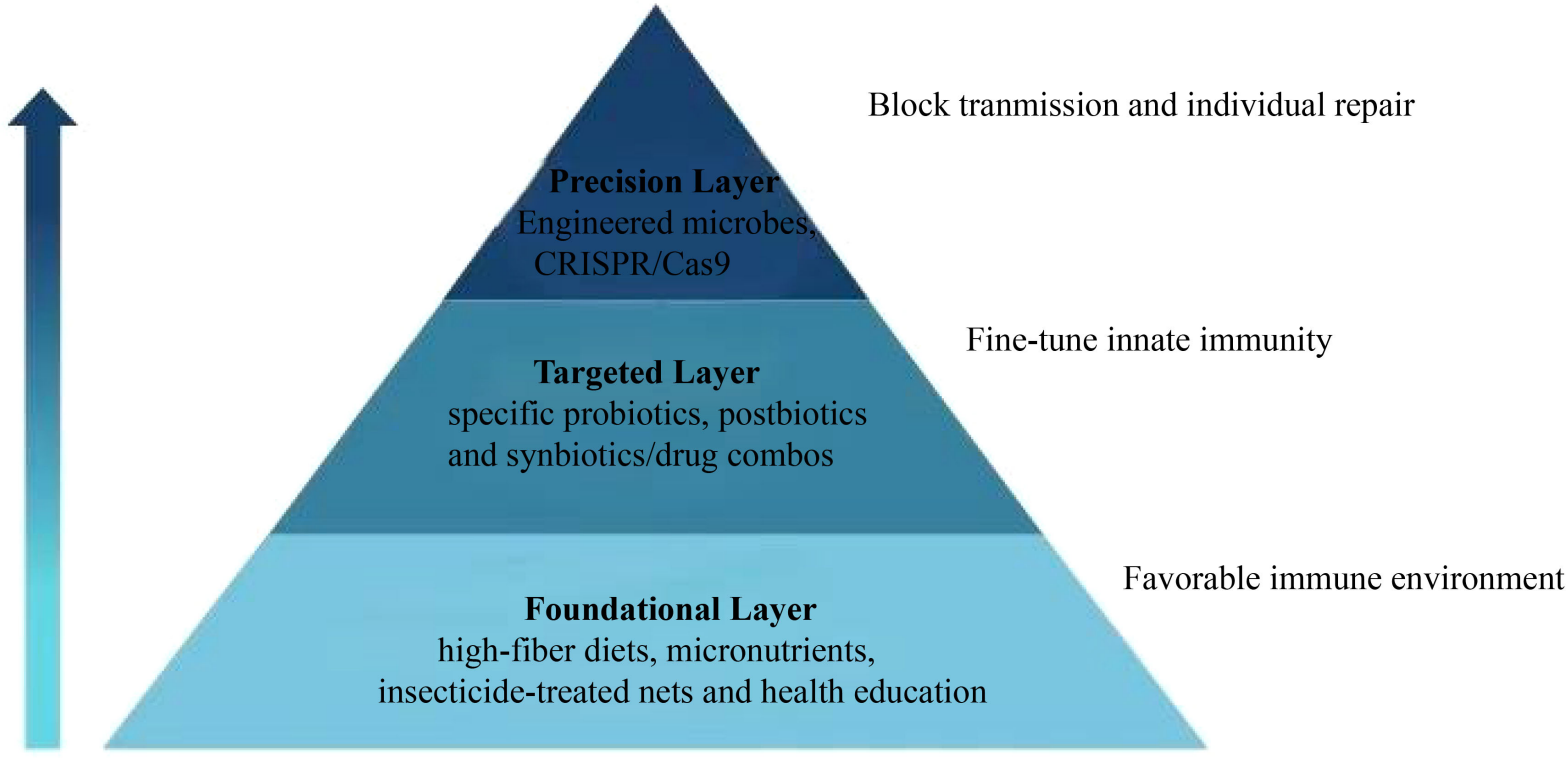
Three-tier precision public health pyramid for malaria control targeting the microbiota-barrier-innate immunity axis.

With this basic overview in mind, we here put forward an integrative and visual three-tier precision public health pyramid model (**Figure 2**) which presents a structured depiction of interventions encompassing foundational nutritional support, microbiota targeting, and “precision” biotechnologies, and diagrams the intervention pathways targeting the “microbiota-barrier-innate immunity” axis. As a transparent roadmap, we believe that our pyramid will ably convey the frontier science and its implications for public health and disease prevention in practice on the ground in Africa-control of poverty-associated disease.

The base tier focuses on ensuring broad disease control goals by bolstering primary health systems and essential interventions around them. At this tier implementation could refer to a constellation of community health volunteers by village which maintains enduring community services and screening for malaria (92). To ensure that quality services are provided, regular supportive supervision, on-the-job, of healthcare facilities must be done. The cost of each supervision ranges from 44 to 333 US dollars. Given the high cost, it may be difficult to cover all institutions. Therefore, internal quality assurance measures are recommended as an alternative or supplementary solution (93).

Supplying probiotics with proven anti-malarial properties represents a promising advanced strategy. Animal studies provide evidence for this approach. In a chloroquine-sensitive *Plasmodium berghei* ANKA mouse model, daily probiotic supplementation clears parasites by effectively suppressing parasitemia and thus improves survival (94). The efficacy of this intervention can be evaluated by its ability to induce specific immune responses including anti-α-Gal antibodies. However, studies have found that the antibody levels induced by the selected lactic acid bacteria are not significant compared with the control strain *Escherichia coli O86:B7 (*95). This indicates that strain screening and optimization remain key targets for future breakthroughs.

The precision tier is concerned with risk management of frontier technologies and the targeted development of community diagnostic capacities. For purposes of gene drive deployment, we assume release will target every member of a given community selected for field tests, with the crucial caveat that broad stakeholder engagement is ensured. Following release, it is vital that long-term monitoring begins on the health impact on people and biodiversity and the efficacy of that technology. Over the course of this monitoring period, the full net ecological and epidemiological impact will occur (96).

## 5 Toward field implementation

### 5.1 Population prioritization and targeted deployment

Infant gut microbiota at baseline varies markedly by geography so any nutritional interventions for infants may need to be locally specific. In a clinical trial of rice bran supplementation in Nicaragua and Mali, where infants in the randomized control groups were of the same age, marked national differences in infant gut microbial community structure were observed. At the national level Mali and Nicaragua differed at the phyla and family level where the infants had different dominant microbial profiles. Following the same rice bran intervention, Nicaraguan infants showed a significant enrichment of taxa with glycolytic and SCFA-producing potential, such as Lachnospiraceae, *Bifidobacterium*, and *Veillonella*. In contrast, *Lactobacillus* was the most prominently responsive genus to rice bran in Malian infants (97). Even within the macro-category of low- and middle-income countries, distinct geographic and socio-cultural environments shape unique gut microbial ecosystems. Therefore, future nutritional interventions, including the application of prebiotics, probiotics, or functional foods, cannot be viewed as universally applicable. A more feasible approach is to develop more targeted nutritional intervention programs. This should be done on the basis of a thorough understanding of the baseline gut microbiota characteristics of the target population. It will help promote the implementation of truly personalized nutrition strategies.

#### 5.1.1 Children’s groups

As far as priority of interventions is concerned, children should be identified as the primary target group, not only because of the disease burden that they bear, but because of the high degree of plasticity in their innate immune system which remains at a critical window of development. In the 2019 transmission season in the District of Dangassa in Mali, extending Seasonal Malaria Chemoprevention (SMC) to school-age children (5–14 years) achieved 96.5% community compliance. The intervention group showed a significant decrease in prevalence of *P. falciparum* infection, the prevalence of *anaemia*, and the cumulative incidence of infection compared with the control group (98).

The selection of probiotic strains must be grounded in robust clinical evidence. Randomized controlled trials have demonstrated that *Saccharomyces boulardii* and a quadruple-strain viable *Bifidobacterium infantis* preparation can effectively prevent antibiotic-associated diarrhea in infants and young children, significantly reducing its incidence and shortening its duration. Furthermore, probiotic administration for 14 or 21 days results in significantly greater improvement in gut microbiota structure compared with a short-term 7-day regimen, more effectively lowering the fecal cocci-to-bacilli ratio (99). In sub-Saharan African settings where malaria and bacterial infections often co-circulate and antibiotic use is frequent, the use of such clinically characterized strains may offer a potential strategy to help preserve gut microbiome integrity and mitigate the worsening of malnutrition. However, direct evidence from local populations remains limited. Further studies are needed to assess whether full-course prophylactic probiotic supplementation during high malaria transmission seasons or alongside antibiotic treatment confers sustained benefits for intestinal homeostasis in children in these specific contexts.

More fundamentally, interventions should consider that age and nutritional status are intertwined. In a longitudinal study in Burkina Faso, a novel aspect of the association between malnutrition and malaria infection emerged that was age-specific. That is, among children aged 0–30 months being heavier and taller was paradoxically associated with greater odds of being infected with malaria (100). This discovery suggests that children in this age group may have a unique immunometabolic susceptibility, and also suggests that young children should not be considered a homogeneous group in intervention design. Age stratification may become an important dimension of consideration. Ensuring the nutritional status of children under 30 months of age may be a necessary foundational measure; if further evidence is obtained to support it, the application of specific probiotic strains may help enhance intestinal barrier function, regulate immune responses, and potentially reduce their risk of susceptibility. For children over 30 months old, interventions may focus more on breaking the vicious cycle between malnutrition and susceptibility to malaria, such as continuously optimizing nutrient absorption through probiotics. However, these age-based intervention hypotheses still need to be rigorously researched and validated in order to provide reliable evidence for practice.

#### 5.1.2 Pregnant women

Pregnant women should also be targeted for adjunctive intervention. Infection with *Plasmodium berghei* on gestation day (GD) 6 in pregnant mice was associated with morbidity, fetal resorption rates, and stillbirth. Infecting pregnant females at the GD10 stage resulted in a greater biting preference of the infected mosquito vectors for the pregnant host at this stage, and higher parasite burdens in the uterus and spleen of pregnant mice and designated tissues, as well as increased corticosterone, decreased progesterone, and systemic immune activation. The dysregulated pathological changes evoked during pregnancy prevented normal fetal growth (101). It should be pointed out that the above findings are based on rodent models and cannot be directly extrapolated to humans, but provide preliminary clues for understanding the potential impact of infection timing and host physiological status on intervention strategies. In early pregnancy, using a low toxicity anti-malarial regimen to control infection remains the top priority; on this basis, the use of low-dose prebiotics such as inulin to moderately regulate the intestinal microenvironment may be an exploratory approach, but its safety and efficacy in human pregnancy still need to be systematically evaluated. In mid-pregnancy, the combination intervention of long-acting insecticidal mosquito nets and functional probiotics may be considered as a potential strategy. Probiotics may help regulate maternal immunity and hormone levels, and may alleviate placental inflammation, thereby reducing interference with vertical transmission of microbiota. However, the above intervention hypotheses still need to be validated through rigorously designed human studies in order to form clinically significant conclusions.

Importantly, evidence shows that intermittent preventive treatment in pregnancy may benefit mothers and infants through multiple non-malarial mechanisms, even in parts of Africa where *Plasmodium falciparum* is highly resistant to sulfadoxine-pyrimethamine(SP) or in non-malaria-endemic areas. Its broad‐spectrum antibacterial activity can suppress genitourinary infections, with a consequent reduction in inflammatory response to infections (102). It is hypothesized that SP may also exert positive effects on the maternal intestinal environment. It could potentially alleviate certain aspects of environmental enteric dysfunction to improve nutrient absorption. However, direct evidence concerning its impact on the maternal gut microbiota is currently insufficient. Therefore, when establishing a three-tiered intervention system for pregnancy in malaria-endemic areas of Africa, intermittent preventive treatment in pregnancy with sulfadoxine-pyrimethamine can be used as an auxiliary intervention with important potential value. It may act synergistically with prebiotics or probiotics. Its unique value deserves attention and further verification, especially in regions with high SP resistance.

To minimize the individual heterogeneity problem in future interventions, there is a great need to move towards precision medicine. Studies carried out on a mother–infant cohort in West Africa have shown that the presence of active placental malaria increased the risk of parasitic infections in nine-month-old infants by 5.8 times. Increasing iron stores in children are significantly correlated with an increased risk of infection whilst chorioamnionitis caused a reduction in risk of childhood parasitosis of 60% (103). This suggests that controlling placental inflammation may have important value in reducing mother-to-child transmission of pathogens. At the same time, it also indicates that in the absence of clear evidence of iron deficiency, routine iron supplementation may lack sufficient basis, and stratified intervention strategies based on infant iron storage status may be more desirable. The use of inducible anti-inflammatory probiotics may also be evaluated for their effects together with a systematic analysis of their long-term uses as far as child health is concerned.

### 5.2 Resource adaptation and implementation pathways

To address implementation barriers of African field situations such as lack of cold chains and high costs, intervention strategies for all levels need to be locally designed. At the basic level, it is possible to consider relying on local agricultural systems to support the cultivation of high fiber crops such as corn and beans, and explore the integration of prebiotic ingredients such as inulin into staple food processing. At a more targeted intervention level, it is possible to try using probiotic strains that produce spores and are heat-resistant, packaged in vacuum aluminum foil; Alternatively, it can be made into oral powder with a simple quantitative spoon to enhance its applicability in hot weather conditions. At the level of precise intervention, it is advisable to prioritize the use of existing infrastructure such as malaria monitoring points for microbial sampling and monitoring, in order to avoid the need for new construction as much as possible; at the same time, developing simplified laboratory processes and portable diagnostic equipment may help reduce technical barriers and operating costs. It should be pointed out that the above strategies are still in the exploratory stage, and their feasibility, acceptability, and potential for promotion still need to be systematically tested and iteratively optimized in practical scenarios.

Community acceptance of novel control tools is critical to overcoming the bottlenecks to their implementation. A cross-sectional study in a health district of Burkina Faso demonstrated that local populations in general possess a basic to good understanding of the transmission dynamics. Over 90% of surveyed individuals reported adopting commonly used malaria control measures. In relation to the larvicide based on *Bacillus thuringiensis israelensis*, it was found that most respondents were aware of its efficacy in eradicating mosquitoes, while there was overwhelming enthusiasm for providing funds for the control measure (104). Therefore, Bti larvicides may be more acceptable to communities and may be suitable for scale-up, but they still require confirmation across additional regions and across multiple time points.

The existing knowledge base of malaria prevention and control in the community may provide some support for introducing new intervention methods. In some cases, demonstrating the practical effects of new tools may be more helpful in enhancing community confidence than simply theoretical awareness campaigns. The conventional application of Bti larvicides, although relatively controllable in single use cost, usually still requires active participation and effective mobilization from the community. On the basis of conducting initial community engagement, it is possible to try to construct a community participation model that adapts to local conditions, and establish a clear correlation between the use of such tools and the specific benefits that the community can obtain. This path may help actively build social license, providing support for the ethical implementation and long-term sustainability of intervention measures. However, it should be pointed out that the design and actual effects of such participation models are highly context dependent, and further exploration and evaluation are still needed in different socio-cultural contexts.

### 5.3 Macro-level support and system integration

In terms of economic feasibility, a modeling study based on the Malaria Strategy (2019–2023) in Kenya showed that although the large government health expenditure of KES 61.9 billion in the first implementation period led to increased production costs and prices for consumers thus putting short-term pressures on household well-being, the overall malaria cases declined by 75%. Thus labor force health improved to the extent that this became a strong driver for net Gross Domestic Product (GDP) growth (105). This finding suggests that the costs of microbiota-based interventions might be framed, at least in part, as an investment in workforce health. By potentially reducing disease incidence, work absenteeism, and productivity losses, such interventions could, under favorable conditions, contribute to longer-term economic returns. This line of reasoning may be particularly relevant for African economies with high labor dependency, though the transferability of this model to microbiota interventions remains hypothetical and requires further empirical validation through field studies and cost-effectiveness analyses in specific national contexts.

To help mitigate the potential dilution of intervention efficacy due to cross-border transmission, there may be a need to strengthen regional coordination and enhance precision surveillance. The importation routes of *Plasmodium vivax* from Central America and *Plasmodium falciparum* from Africa were successfully traced by Mexico by establishing a clear definition of imported malaria cases, verifying pathogens using thick blood films and molecular biological techniques, and considering case characteristics and migration routes. This yielded vital evidence for the targeting of control (106). The problems facing Africa, with its high incidence of cross-border population movements, might be consequently two-fold, being those of the danger of transmission of endemic types from the immigrant population to the rest of Africa, and the importing of foreign *Plasmodium* strains. In this context, it may be considered to explore the establishment of standardized health monitoring points in border areas of African countries. However, the feasibility and actual effectiveness of such measures highly depend on local infrastructure, resource allocation, and cross-border collaboration mechanisms. Conducting health assessments for mobile populations may involve the detection of gut microbiota and innate immune-related biomarkers. If timely and tailored intervention measures can be combined, it may help reduce the risk of cross-border transmission of malaria parasites. However, it should be pointed out that this idea is still a theoretical concept, and its operational feasibility, predictive validity, and actual public health value still need to be systematically evaluated through rigorous on-site research.

### 5.4 Technical support systems for field implementation

#### 5.4.1 AI risk prediction and point-of-care diagnostics for empowering targeted interventions

Integrating and analyzing multi-source data for malaria risk prediction could facilitate more precise and targeted preemptive allocation of intervention resources. A predictive model based on a combination of Random Forest (RF) and K-means spatial clustering technologies successfully predicted weekly malaria case incidence with a high degree of accuracy in the Legal Amazon region of Brazil (107). This hybrid modeling approach, which combines machine learning models with spatial clustering, provides a promising tool for enhancing malaria surveillance and guiding more effective public health strategies, especially in malaria control efforts in high-risk areas.

This optimization scheme makes the detection of malaria parasites relatively faster in primary healthcare scenarios, and in principle supports the automation of personalized intervention plans, which may help build a more integrated “diagnosis-decision-intervention” loop. Sukumarran et al. pruned the C3–C5 residual blocks in the backbone network, removing redundant structures, and further replaced the CSPDarknet53 backbone with the lighter ResNet-50. The resulting lightweight model, YOLOv4-RC3_4, achieved higher detection accuracy on the independent Dataset B compared with the original YOLOv4 model. This optimized model maintains high detection accuracy while significantly reducing giga floating-point operations and model size, and is minimally affected by false-positive predictions (108). The above feature suggests that such models have the potential to be embedded in portable diagnostic devices, which may enhance their applicability for real-time detection in resource-limited environments in Africa. If infected individuals can be quickly identified through such devices, it is possible to achieve automatic matching of personalized immune intervention strategies and provide support for real-time adjustment of treatment plans. However, integrating automated decision support systems into field deployable diagnostic tools is still in the early stages of exploration, and its actual potential in improving intervention efficiency requires further technological development, clinical validation, and adaptive optimization in specific contexts to obtain reliable evaluations.

#### 5.4.2 Mosquito blood meal source analysis enables precision blocking of transmission chains

To facilitate the directed deployment of insect-specific interventions such as the release of modified strains of the bacterium described in the precision tier, it may be important to know at least the blood meal sources of the mosquitoes to monitor transmission chains. The technology we need for this is Matrix assisted laser desorption ionization time-of-flight mass spectrometry (MALDI-TOF MS) protein fingerprinting. A newly developed application of this technology enables the efficient identification of mixed blood meals and the last blood meal from *Anopheles gambiae*, *Anopheles coluzzii* subspecies, and *Aedes albopictus (*109).

This method has to some extent broken through the limitations of traditional PCR technology in mixed blood meal analysis, and may provide support for complex human–mosquito–animal multi-host interactions in African malaria-endemic areas, helping to identify key reservoir hosts. If effectively validated under field conditions, this technology is expected to help map more detailed regional malaria transmission networks and assist in locating high-risk vector and host populations. This type of data can provide core support for the design of future targeted intervention strategies, such as phage intervention or specific vector control measures, and may drive the exploration of precise public health models aimed at blocking the transmission chain. However, the reliability, scalability, and cost-effectiveness of this technology in real-world environments still require further on-site validation and implementation studies to evaluate.

### 5.5 Frontier technologies awaiting breakthroughs

#### 5.5.1 Development and application of region-tailored phage therapy

The midgut and salivary gland microbiota of *Anopheles sinensis* mosquito populations in South Korea’s high- and low-malarial endemism areas are distinct. The high-endemicity area harbored 17 unique OTUs, while the low-endemic area was completely dominated by *Pseudomonas* (110). This discovery suggests that if further validated, the development of region-specific phage libraries for pathogenic OTUs related to high prevalence areas may be considered to minimize the uncertain effects of non-specific interventions. However, the universality of the microbial distribution pattern in a wider geographical range, as well as the feasibility, specificity, and ecological safety of such phage intervention strategies, still requires further research and evaluation.

Phages have the potential to act as ecological regulators in mosquito larval breeding waters. Studies show that phages targeting bacterial genera closely associated with larvae, such as *Enterobacter* and *Pseudomonas*, can alter the aquatic microbial community, which in turn affects larval development and survival. Moreover, phage combinations can exert synergistic effects (111).A variety of core bacterial genera have been successfully isolated from the midguts of mosquitoes, including *Microbacterium, Enterobacter, Klebsiella*, and *Pantoea*. Twelve lytic phages capable of lysing *Enterobacter*, *Klebsiella*, and *Pantoea* have been identified. Their genomes contain no antibiotic resistance genes or virulence genes, indicating favorable biosafety (112). Accordingly, advancing the development of phages that target and regulate the midgut microbiota of adult mosquitoes has become an urgent research direction for further exploration.

Our approach must break free from the traditional restrictions imposed on investigations into gut flora, which only concern the midgut. Through comparative analysis of the microbiomes of Aedes aegypti and Aedes albopictus, this study found that both gut and systemic microbiota of these mosquito species are dominated by Proteobacteria and Methylobacterium as core dominant groups. However, significant differences exist in the composition of their systemic microbiota, suggesting that host species is one of the key factors shaping the structure of microbial communities. Functional prediction analysis further reveals that the microbiota of these two mosquito species differ significantly in specific functional pathways, and such differences are particularly prominent in the systemic microbiota (113). These results support the inference that the assembly of adult gut microbiota may be driven mainly by shared environmental factors, while the composition of whole-body microbiota is more strongly influenced by host genetic background.

Most of the studies above used 16S rRNA gene sequencing to describe structural differences in vector microbiota. This method is valuable for detecting uncultured or difficult-to-culture bacteria, but its limitations for translation need careful consideration (114). One prospective preclinical study reported that, when the final diagnosis was used as the gold standard, the sensitivity of 16S rRNA sequencing was only 38.3% (115). In addition, this approach usually has limited resolution between closely related species and cannot directly indicate the functional state of the microbiota. Tools such as PICRUSt2 attempt to infer functional profiles from 16S sequences. However, a systematic benchmarking study has shown that inferred functions can differ substantially from empirically measured metagenomic data and may lack the sensitivity required to capture key health-related functional shifts in the microbiome (116). Therefore, core genera identified from these data should have their true transmission-promoting or antimalarial roles confirmed with multi-omics strategies, including microbial cultivation, metagenomics, and metabolomics.

By analyzing the microbiomes of African malaria vectors *Anopheles arabiensis* and *An. funestus*, the researchers evaluated the effectiveness of two low-cost preservation methods, silica gel desiccant and RNAlater. Microbial diversity was not affected by preservation method, time, or mosquito gender, but the type of detection technology significantly influenced the analysis results. Silica gel preservation was suitable for subsequent culture-dependent microbial analysis. RNAlater preservation was suitable for subsequent culture-independent molecular analysis (117). This result provides an economical, effective, and reliable sample preservation and processing option for mosquito-borne microbiome research under resource-limited conditions, broadening the technological path for downstream analysis of field samples. Future research should focus on functional verification of core bacterial strains, ecological dynamic monitoring, and establishment of standardized procedures. This preservation-analysis strategy is expected to be extended to other vector insects, providing methodological support for global infectious disease prevention and control research.

#### 5.5.2 Complement inhibitors enhance the safety of novel interventions

Small molecule complement inhibitors may serve as an important safety supplement to precision-level intervention strategies, having the potential to mitigate the risk of hypersensitivity reactions related to complement activation that may be encountered with emerging approaches such as bacteriophage therapy and nanovaccines. Furthermore, these inhibitors also contribute to enhancing the hemocompatibility and immunocompatibility of nanoparticles in various animal and even human donor samples. CR2-CR1 Fusion Protein (CR2–CR1) manifests an unusually high degree of versatility and power, which, among other things, inhibits complement activation in most nanoparticles in human serum and plasma. It also efficiently blocks the immune uptake of many classes of nanoparticles in the human blood, making it a candidate for the more universally applicable inhibitors. On the other hand, iptacopan has striking advantages in the cross-species category in that it can block the complement cascades activated by nanoparticles in human serum as well as in several species including mice, rats and dogs. Accordingly, it decreases complement opsonization and immune cell uptake. In addition, it also significantly decreases acute infusion reactions, after nanoparticle injection in rats and dogs, but has donor-dependent variability and its versatility and power are not up to those of CR2–CR1 (118).

Complement inhibitors, especially broad-spectrum and potent inhibitors like CR2-CR1, are expected to serve as a universal strategy to improve the safety of nanomedicines. Iptacopan is currently the most translationally promising candidate drug owing to its clinical availability and cross-species efficacy, although its effects may vary across individuals and nanoparticle types. Future studies must concentrate on clarifying the differential impact these inhibitors may have in populations in malaria-endemic areas to identify patients that are at a high risk for complement activation. This may help achieve a more accurate balance between efficacy of intervention or safety allowing a more rigorous foundation of the large-scale application of novel interventions in African malaria field situations. There is also the need for investigations of safety and efficacy of application of these agents in endemic populations prior to their application in malaria control, and furthermore, problems of cost and storage and ease of utilization must be addressed.

### 5.6 Economic feasibility of the three-tier intervention framework

In accordance with the WHO Global Malaria Technical Strategy, about 8.7 billion US dollars will be needed annually by 2030 for malaria control and elimination to meet the core targets under the strategy (119). The three-tier intervention platform proposed here offers a graduated approach, ranging from low-cost, broad-based interventions to high-investment precision technologies. This framework aims to maximize public health impact within constrained donor budgets and provides a translational roadmap toward the global goal of reducing malaria case incidence and mortality by at least 90% by 2030.

Inulin has been chosen as a model treatment for the foundation tier. This combination of low cost and health benefits offers significant cost savings and broad utility. Inulin is a natural fiber present in thousands of plant species (chicory root, Jerusalem artichoke), so supply is ample. As a high-quality prebiotic, inulin can promote the growth of beneficial microbiota and enhance intestinal barrier function. It also improves the absorption of nutrients including calcium and iron (120). These effects may provide an immunological basis for preventing Plasmodium infection, relieve malnutrition in many African children, and thus target a key risk factor of severe malaria. Therefore, inulin may help realize low-cost malaria prevention and control, while providing diverse synergistic health benefits. This further supports its potential role as a core intervention for populations at the bottom of the pyramid.

Drawing on existing research evidence regarding probiotic applications, we hypothesize that certain probiotics used for the adjunctive prevention and treatment of malaria may also demonstrate considerable cost-effectiveness and mechanistic feasibility. Taking North America as an example, preventing antibiotic-associated diarrhea with specific probiotics such as Bio-K+ CL1285 or LBC80R could reduce per capita medical costs by US$1,968–2,661 (121).Though this research field is dissimilar to malaria prophylaxis, it demonstrates the ability of probiotics to lower rates of secondary infections and healthcare requirements. Study on probiotic-aided oral immunotherapy (PPOIT) for peanut allergy in children shows that while this therapy entails higher initial medical costs, its substantial net health benefits result in a cost-effectiveness ratio that is far superior to traditional cost-effectiveness thresholds (122). This implies that when considering the economic impact of probiotic intervention strategies at the second level, we should be looking at their health benefits over the longer term and total effect on overall healthcare expenditure, thus giving a fuller picture of their costs and feasibility for use in the field in Africa.

Within that tier, intervention methods such as gene drive technology are rational long-term investment directions. While the upfront cost will be high, the real value lies in the potential to break the chain of transmission of malaria at source, for good. According to a systematic review, when still at the malaria elimination phase, the cost of surveillance–systems for epidemics could then be as high as US$389.82 per case averted (123). Relative to this ongoing cost of maintenance, a one-off investment on a frontier technology capable of delivering eradication might have a higher ex post return on investment.

### 5.7 Translational bottlenecks

#### 5.7.1 Complexity of mechanistic understanding

The same applies to attempts to simply boost beneficial metabolites like the SCFAs. While there is plentiful evidence that these compounds serve to protect immune homeostasis and barrier function, it is a fallacy to think of them as universally beneficial molecules, and a recent study in certain mouse models shows that fecal propionate levels are higher in disease-susceptible individuals. Furthermore, it reveals no robust correlation between malaria severity and levels of fecal propionic acid (124). This paradoxical phenomenon reveals the highly context-dependent functionality of SCFAs.

This contradiction is not unique. It could be that systemic dysbiosis of the microbiota leads to excessive propionate production, which competitively inhibits butyrate from binding to their shared receptors thereby subverting the anti-inflammatory ascribed roles of butyrate. Or perhaps as with malaria-dysregulated intestinal barrier leakage. SCFAs are thus “lost” before they have the opportunity to enter the systemic circulation. SCFA concentrations measured from feces reflect the actual production within the colonic lumen, but not necessarily their bioavailability in the important immune organs or circulation. Alternatively, it may be that in a highly inflammatory environment the capacity of SCFAs to metabolically reprogram particular immune cells may be compromised, unintentionally enhancing pro-inflammatory responses.

In the context of gut inflammation, however, the SCFA receptor FFAR2 takes on a completely different signaling role. Under healthy, homeostatic conditions, signaling through FFAR2 promotes immune homeostasis and prevents neuroinflammatory genes from being expressed, whereas in a dextran sodium sulfate (DSS)-induced model of colitis, FFAR2 knockout downregulated inflammatory genes in microglia. Long-term inulin supplementation served to exacerbate inflammation rather than ameliorate it (125). This may mean that under systemic inflammation, SCFAs may shift from inflammation suppressors to potential facilitators of further pathology. The immunomodulatory benefits of dietary fiber have a critical tipping point depending on the inflammatory milieu. When the host is in an acute or dysregulated immune state, nutritional interventions to ramp up SCFA production may fail to escort the immune system back to homeostasis, serving to strengthen pathological immune response instead. The success of SCFA theory-based interventions is therefore highly unpredictable and bi-polar in its effects depending on the nuances of the individual host’s immune status.

#### 5.7.2 Individual heterogeneity

Individual heterogeneity in response to probiotics can be partly explained by the strain dependence of therapeutic efficacy. Evidence from a meta-analysis on chronic constipation supports this mechanism: *Bifidobacterium lactis* exerts a significant effect, whereas probiotic mixtures, *Bacillus coagulans* Unique IS2, and *Lactobacillus casei* Shirota show no such effect (126). This strain specificity may be a key factor contributing to variability in clinical outcomes. Certain probiotics may improve treatment response, stool frequency, and overall constipation symptoms, providing cautious optimism for their use as dietary management strategies. Insufficient evidence currently supports the use of synbiotics for the treatment of chronic constipation. Caution is warranted in interpreting these findings given the high heterogeneity across studies and the risk of bias.

In the host intestine, the colonization fitness of probiotics also faces an ecological barrier mediated by the resident microbiota. There is evidence that even when the same multi-strain combination is given, individuals show person-, region- and strain-specific mucosal colonization patterns and that exogenous probiotics have difficulty achieving stable colonization of ecological niches that are already frequently occupied by the resident microbiota (127). Consequently, the long-term benefits of probiotics are often limited. Therefore, current probiotic intervention strategies still face challenges regarding their persistence and stability.

#### 5.7.3 Inherent risks of the technology itself

Gene drive technology has self-propagating characteristics. This makes its ecological outcomes hard to predict, with ramifications that could be substantial and irreversible to non-target species and ecosystems (128). To address this risk, working groups have established phased testing pathways for gene drive mosquitoes, aiming to manage risk through gradual increases in environmental and human exposure (129). However, these efforts are not yet fully matured, and a globally binding international regulatory framework is sorely lacking, creating an ever-increasing divide between the development of technology and the management of risk.

Proponents of gene drive field research can draw on the experiences from dengue eradication field trials, cluster randomization trials, and pragmatic clinical trials to obtain guidance on managing the informed consent application and participation process for individuals in the trial communities. The social enablers for deployment of gene drive technology are deeply challenging, with one major hurdle being widespread public distrust of genetically modified organisms and the effective impossibility of receiving everyone’s informed consent within a region that considered itself “field trials” (130). The basic contradiction here is a core ethical dilemma for technological translation. This requires building social license, often by crafting ongoing transparency, meaningful benefit-sharing and a genuinely visible responsiveness to concerns of communities.

#### 5.7.4 Safety risks of fecal microbiota transplantation

In two independent clinical trials, two patients developed Escherichia coli bacteremia producing extended-spectrum beta-lactams (ESBL) after receiving oral capsules of fecal microbiota transplantation (FMT) from the same donor, and one patient died. Whole genome sequencing confirmed that the infecting strains of two patients were clonally related to the strains in the donor’s feces. After analysis, it was found that all FMT capsule preparations of the donor tested positive for ESBL in microbiological screening. This led the US Food and Drug Administration (FDA) to issue a nationwide safety alert and require testing for extended spectrum beta lactases as part of donor screening (131). It exposes a fundamental flaw in the current practice of FMT. Careful donor screening and the development of permanent pathogen surveillance systems are the minimum requirements in terms of risk mitigation for recipients, continuing to challenge recent regulatory efforts.

The application of FMT remains largely empirical, as our understanding of its precise mechanisms of action is still limited (132). Furthermore, feces themselves constitute a highly complex and variable matrix containing thousands of microbial species, viruses, fungi, and metabolites (133). This method leads to huge variability between different FMT products and their batches even when derived from the same donor. However, pharmaceuticals and other conventional medicinal products require the development and regulation of consistent quality and control. The high variability between FMT products poses a significant challenge to its standardized production and reliable assessment of efficacy.

#### 5.7.5 Implementation barriers in African field settings

Cross-border movement of people is a constant source of imported malaria cases. Border areas also have the added difficulty of uncontrolled cross-border movement coupled with other challenges such as human resource shortages and border permeability (134). Consequently, the effectiveness of community-based interventions may be diluted by the persistent influx of imported infections.

In many under-resourced areas, the cold chain presents significant challenges to the routine vaccine management process. The percentage of health workers who have sufficient knowledge regarding the management of the vaccine cold chain in Ethiopia is 49.92% (135). The observed rate of proper practice is even lower, with only 50% of health centers meeting the required standards (136). This poses a significant challenge for the field application of probiotics requiring cold storage and for future biological agents.

#### 5.7.6 Progress and limitations in methods for vector microbiota research

Evidence suggests that during DNA extraction, protocol choice significantly influences DNA yield and microbial community recovery; for example, the AllPrep DNA/RNA Mini Kit can increase DNA concentration and measured microbial diversity (137). Protocols that add a mechanical bead-beating step can also substantially improve DNA quantity and microbial diversity (138). This means that without standardized and optimized pre-processing workflows, the vector microbial community profile obtained may deviate from the real situation and can directly bias later association analyses with malaria transmission phenotypes.

16S rRNA gene–based amplicon sequencing is still widely used, but primer selection can create amplification biases across bacterial groups, and some taxa may be underdetected or missed. Even so, available studies indicate that the main biological signals stay consistent across different primer sets, supporting the view that 16S sequencing is generally robust for identifying major between-group differences. The same work also stressed that metagenomic sequencing offers finer taxonomic resolution than 16S sequencing and recommended combining multiple technologies to achieve a more complete community characterization (139).

Currently, sequencing-based fecal microbiome studies usually report microbial taxa and metabolic pathways as relative fractions of the sequence library generated in each run. Although relative profiling can capture disease-associated microbiome variation, it is limited for explaining how the microbiota relates to host health in quantitative terms. Comparisons based only on relative data cannot specify the magnitude or direction of true changes in taxon abundance or metabolic potential. When microbial loads vary substantially between samples, relative profiling makes it harder to connect microbiome features with quantitative variables such as physiological parameters or metabolite concentrations (140).

Multi-omics methods are increasingly driving this field forward. Metagenomics avoids PCR amplification, can deliver more accurate species identification, and directly shows the functional gene repertoire present in microbial communities, allowing inference of functions such as resistance genes. However, metagenomics still faces limitations, including difficulties in sequence assembly, challenges in stitching high-repeat regions, and database biases that favor known cultivable species (141).

At the same time, metabolomics can provide more direct chemical support for “microbiota–immunity” interactions by systematically measuring small-molecule metabolites produced by microbes. Yet metabolomics itself involves multiple practical challenges, including analytical standardization, identification and quantification of complex metabolites, and interpretation of large-scale datasets (142). Accordingly, effective translation depends on rigorous study design, standardized workflows, and careful integration with other omics layers.

#### 5.7.7 Remaining obstacles in engineered microbiome modulation

Xia et al. summarized that current engineering strategies mainly fall into physicochemical engineering and genetic engineering. Physicochemical engineering increases functional diversity by linking bacteria with abiotic systems and can avoid genetic instability, but material safety and controllable degradability remain central issues. Genetic engineering can precisely introduce new genetic functions into bacteria; however, when multiple genes are integrated, off-target effects and genetic contamination risks can occur. In addition, engineered bacteria still face a long path toward clinical translation: safety concerns have partly slowed approvals, and it is difficult to achieve specific, precise, and controllable bacterial proliferation and dissemination within target regions. If innate immune responses and cytokine storms are not adequately reduced, treatment safety is further compromised. Moreover, the complex microenvironment and pathogenic mechanisms within a single lesion can make it refractory and highly drug-resistant, and currently, engineered-bacteria approaches still lack sufficient practical evidence and theoretical foundations for broad clinical use (143).

For field intervention routes, the use of *Wolbachia* in *Aedes* mosquitoes for viral disease control provides an informative reference. An eight-year monitoring program reported that in *Wolbachia*-infected *Aedes aegypti* populations released in the Cairns region of Australia, the symbiont genome remained highly stable, and no adaptive mutations that could weaken viral suppression were detected (144). This experience supports optimism for symbiont-based antimalarial approaches and suggests that, after careful screening and modification, microbes may maintain long-term genetic and functional stability. However, *Wolbachia*’s success should not be directly assumed for other microbial systems in antimalarial settings. For each candidate engineered microbe, long-term stability should be tested over multiple generations in semi-field environments and across different ecological zones.

### 5.8 Future directions

#### 5.8.1 The path to deep analysis of mechanism cognition

To disentangle the unresolved elements of functional directionality and vague thresholds of metabolites such as SCFAs within inflammatory microenvironments, research could begin by performing prospective human cohort studies that collect host plasma, fecal and intestinal mucosal samples during various immune states. Using metabolomics and single-cell transcriptomics, one could map the tissue distribution and bioavailability of SCFAs and other metabolites, clarifying the inflammatory thresholds and local microenvironmental conditions that determine their functional transitions. Additionally, there is an urgent need to intimately grasp the causal nature of intra-axis elements through interdisciplinary approaches to tackle this multidimensional task using pioneering technologies: such as organoid-microbe co-culture systems, humanized animal models, multi-omics; to grasp how exactly microbial metabolites calibrate innate immunity via macromolecular networks. For example, might SCFAs be co-regulators of intestinal function and systemic immunity through neuroendocrine signaling pathways such as serotonin? Finding answers to such questions could lay the theoretical groundwork for discovering novel intervention targets.

#### 5.8.2 Precise matching strategy for individualized intervention

To further explore the relevant research mechanisms and alleviate the problems of significant differences in the effectiveness and colonization resistance of probiotics among individuals, a “probiotic matching algorithm” may be constructed based on the baseline characteristics of individual gut microbiota. By using metagenomic analysis to obtain information on the structure, functional genes, and metabolic potential of individual microbiota, it is expected that machine learning models can be used to predict the degree of adaptation between exogenous strains and host-specific microbiota, as well as their colonization ability and immune regulatory potential.

A novel methodological framework has been proposed, which integrates artificial intelligence and pharmacological modeling techniques, aiming to establish the association between host genetic characteristics and drug response. This framework can identify drug–genome combinations to optimize pharmacogenetic-based treatment regimens with pharmacogenetic significance, and focus on improving treatment strategies for malaria, tuberculosis and other infectious diseases in African populations (145). This approach may potentially promote the development of malaria prevention and control methods, gradually shifting from standardized group intervention paradigms to individualized precision medicine.

#### 5.8.3 Governance of technical risks and trust building

In parallel to progressing personalized interventions, we must also work to actively mitigate the risks of frontier technologies. For ecological risks and ethical dilemmas stemming from technologies such as gene drive, prioritizing international regulatory frameworks and risk assessment systems early on, alongside research into community-participatory governance models, would be prudent. Community acceptance and behavioral factors are often the final arbiter of intervention success. Any novel intervention strategy being proposed must incorporate community engagement, socio-behavioral research and benefit sharing into the core tenets of that research and implementation. Pre-emptively building social license through transparency and benefits sharing will also inform our strategies to tackle the social distrust seen for genetically modified organisms.

#### 5.8.4 Safety and standardization transformation of microbial transplantation

Microbiota transplantation strategies require similar standardization. To avoid infection and batch-to-batch variability the infection risk associated with FMT, donor screening and donor stool processing and pathogen screening must be standardized. Transplantation of whole-stool must shift towards the use of defined microbial consortia/metabolite-based preparations. Matched to metagenomics and metabolomics, defined core combinations of bacterial strains with well-established immunomodulatory functions can be screened to give the product consistency and safety, and reproducible efficacy. This gives them an evidence base for indications where their use might be justified in extreme cases such as severe malaria with microbiota depletion.

#### 5.8.5 Localized integration path for onsite implementation

As technologies converge further, overcoming the implementation hurdles in African field settings is the next frontier. Addressing challenges such as cold chain gaps and cross-border transmission, while strengthening primary health care, represents a two-pronged approach to accelerating the localization and customization of technologies and diagnostics. The development of customized intervention tools based on the local microbial communities is critical. For example, screening and preparing specific phage libraries tailored to the unique midgut microbiota of Anopheles mosquitoes in different endemic regions is a good primer. In diagnostics, focus on next-generation reagents. The use of novel binding agents such as those drawn from sharks, the heat-stable Variable New Antigen Receptor (VNAR) antibody fragments, holds promises for lengthening the shelf-life and making malaria rapid diagnostic tests more field-ready in high-temperature conditions (146). The performance of malaria automatic diagnosis technology based on deep learning models still has room for improvement (147), in order to achieve comprehensive and rapid malaria screening at the primary healthcare level. Meanwhile, utilizing machine learning models to integrate clinical and laboratory parameters may effectively assist in distinguishing malaria from other common tropical infections (148).

#### 5.8.6 Clinical validation and integration of collaborative intervention

These efforts should focus on integrating these synergistic interventions to improve health. The major task ahead lies in the clinical translation of synergistic strategies, with a particular focus on designing exemplary clinical trials that demonstrate the synergistic impact on patient outcomes for cross-tier, multi-modal interventions. We have high hopes in this area for the concept of combining specific probiotics with our antimalarial drugs as a forefront treatment strategy. The probiotics might help to rapidly clear parasites, modulate the microbiota–immune inflammatory axis, establish longer-lasting immune memory, and alleviate pathology. We take our encouragement from successes in other infectious diseases, such as the case where the combination of probiotics with doxycycline for treatment of rosacea not only helped alleviate skin inflammation via the gut-skin axis, but also reduced the abundance of tetracycline resistance genes (149). Similarly, in a toxoplasmosis model, Lactobacillus probiotics combined with trimethoprim-sulfamethoxazole demonstrated significant synergy, greatly enhancing mouse survival and IFN-γ immune responses (150).

Even at the transmission-blocking stage, given the growing challenge of insecticide resistance, we may explore synergistic combination strategies such as “long-lasting insecticidal nets + phage-based larval control” for targeted management of mosquito vectors. Developing novel, eco-friendly control strategies using the mosquito microbiome holds promise; in particular, phages that target insecticide-degrading bacteria including *Lysinibacillus* and *Klebsiella* can be employed. Such approaches are referred to as “symbiotic control” (151). This strategy aims to modulate the gut microbiome of mosquitoes during early larval stages, potentially increasing mosquito susceptibility to long-lasting insecticidal nets and helping establish a transmission-blocking framework in areas with high resistance levels.

## 6 Conclusion

This review aims to build the “microbiota-barrier-innate immunity” axis in a systematic way to construct a cohesive theoretical framework for understanding malaria pathogenesis and intervention design. We outline the key mechanisms whereby *Plasmodium* infection may lead to axis disruption, leading to gut dysbiosis, barrier dysfunction, and innate immune dysfunction which lead to onset and transmission of disease. Using this framework, we suggest a three-tiered intervention pyramid of Foundational, Targeted and Precision, as a treatment strategy that may be useful for African field conditions. This combines classical intervention methods such as insecticide-treated bed nets with microbiota intervention and bioengineering techniques to provide a logical pathway for transition of research from basic science to field trial.

Nonetheless, applying this comprehensive model systematically creates different challenges. Often the mechanistic insights are derived from animal models or only small cohorts in humans, and these have yet to be validated in terms of their generalizability in malaria endemic scenarios in large studies. Moreover, the actions of metabolites, such as SCFAs, are both type-specific and micro-environmental, and the concentration-effect relationships are extremely complex, and the use of fecal concentrations as biomarkers could be misleading. Thirdly, the majority of cutting-edge technologies, for example, engineered bacteria, phage therapy and gene drives, are still in laboratory studies and have no data for the specific applications in malaria. The field safety and ecological risks, as well as cost analysis of the various products, require systematic evaluation. Finally, the synergistic effects of different levels of intervention have yet to be defined and substantial empirical data will be required to identify optimal combinations of integrative diets, probiotics and precision biological sciences.

Future work should be more cross-disciplinary. Future research should promote the axis conceptual framework and the deep integration of interdisciplinary approaches. First, human birth cohorts should be started in areas of highest transmission in Africa, following children from birth to 5y to obtain gut microbiota, plasma metabolites, innate immune cell dynamics and malaria infections (among other things). When these data are combined with metagenomic analysis and single-cell immune profiling, we should begin to see part of the dynamic “microbiota-barrier-innate immunity” axis from 0-5y and how it’s modulating susceptibility to malaria. Second, well-designed randomized controlled trials should focus on specific probiotics or prebiotics. Targeting children in the pre-seasonal setting and pregnant women in the pre-malarial season for gut barrier function, and innate immune response, then relating this closer to clinical malaria incidence. This would provide evidence for the nutrition-immune interventions we are trying to develop for the endemic population. Finally, organoid-microbiota co-culture systems derived from African gut epithelia combined with CRISPR interference may allow modelling of the distinct host-microbiota interactions characteristic of high-transmission settings *in vitro*. This would enable dissection of the drivers/mechanisms whereby SCFAs, cGAS-STING et al, maintain the homeostatic states of the axis compartments to begin understanding the details. In combination these initiatives should take us from an empirical experience-based malaria control paradigm to one entering a novel state of precision Public Health with defined mechanisms, populations and targets of intervention.

We advocate for globally coordinated efforts to: a) establish open-access biorepositories of multi-omics data including microbiota data from different human populations and mosquito vectors in different malaria endemic sites, b) enable mechanistic studies, e.g., organoid-microbe co-cultures, to move from correlation to causation, and c) take adaptive designs for clinical trials which entail a rapid turnover of biomarker information as an exemplar of types of studies for the development of individual interventions.

Through these integrative approaches, we may transform the current paradigm of malaria control into one of precision public health based on a novel framework of “microbiota-barrier-innate immunity” ultimately providing exploitable eco-immunological routes to achieve the ultimate goal of global elimination of malaria.
